# Rolling‐Enabled Nonplanar Heterogeneous Integration for Polarization‐Diverse Nanolasers on Silicon Photonics

**DOI:** 10.1002/nap2.70284

**Published:** 2026-09-05

**Authors:** Byoung Jun Park, Dongmin Shin, Aran Yu, Byeong Uk You, Jaeho Lee, Sang‐Wook Han, Seung‐Woo Jeon, Dongyeon Daniel Kang, Myung‐Ki Kim

**Affiliations:** ^1^ KU‐KIST Graduate School of Converging Science and Technology Korea University Seoul Republic of Korea; ^2^ Physics & Informatics Laboratories NTT Research Inc. Sunnyvale California USA; ^3^ Center for Quantum Technology Korea Institute of Science and Technology Seoul Republic of Korea; ^4^ Division of Quantum Information KIST School Korea University of Science and Technology (UST) Seoul Republic of Korea

**Keywords:** heterogeneous integration, nonplanar integration, photonic crystal nanolasers, silicon photonics, transfer printing

## Abstract

The continued advancement of silicon photonics increasingly relies on heterogeneous integration beyond monolithic fabrication, with transfer printing emerging as a powerful strategy for deterministic and high‐precision assembly. However, existing transfer‐printing strategies remain fundamentally limited to planar integration, restricting access to orientation‐dependent optical functionalities. Here, we introduce a rolling‐enabled transfer‐printing strategy that enables deterministic nonplanar heterogeneous integration through precise mechanical orientation control at the nanoscale. By exploiting rolling‐induced rotation during assembly, identical photonic‐crystal nanobeam lasers are integrated onto silicon waveguides in planar or orthogonal configurations, enabling selective coupling to TE‐like or TM‐like modes without modifying the cavity design or epitaxial structure. Both configurations exhibit stable lasing and polarization‐resolved coupling to silicon waveguides, in good agreement with numerical simulations, confirming that mechanical orientation alone can define optical functionality. By establishing orientation control as a new degree of freedom in heterogeneous integration, this work elevates transfer printing from a placement technique to a functionality‐defining platform, opening new opportunities for compact, multifunctional, and reconfigurable photonic systems on silicon.

## Graphical abstract

Rolling‐enabled transfer printing introduces mechanical orientation as a new degree of freedom for heterogeneous silicon photonics. A conventional TE‐like photonic‐crystal nanobeam laser is mechanically rotated by 90° to selectively couple with the TM‐like mode of a silicon waveguide, enabling polarization‐diverse functionality without redesigning the cavity and opening up new possibilities for three‐dimensional photonic integration.

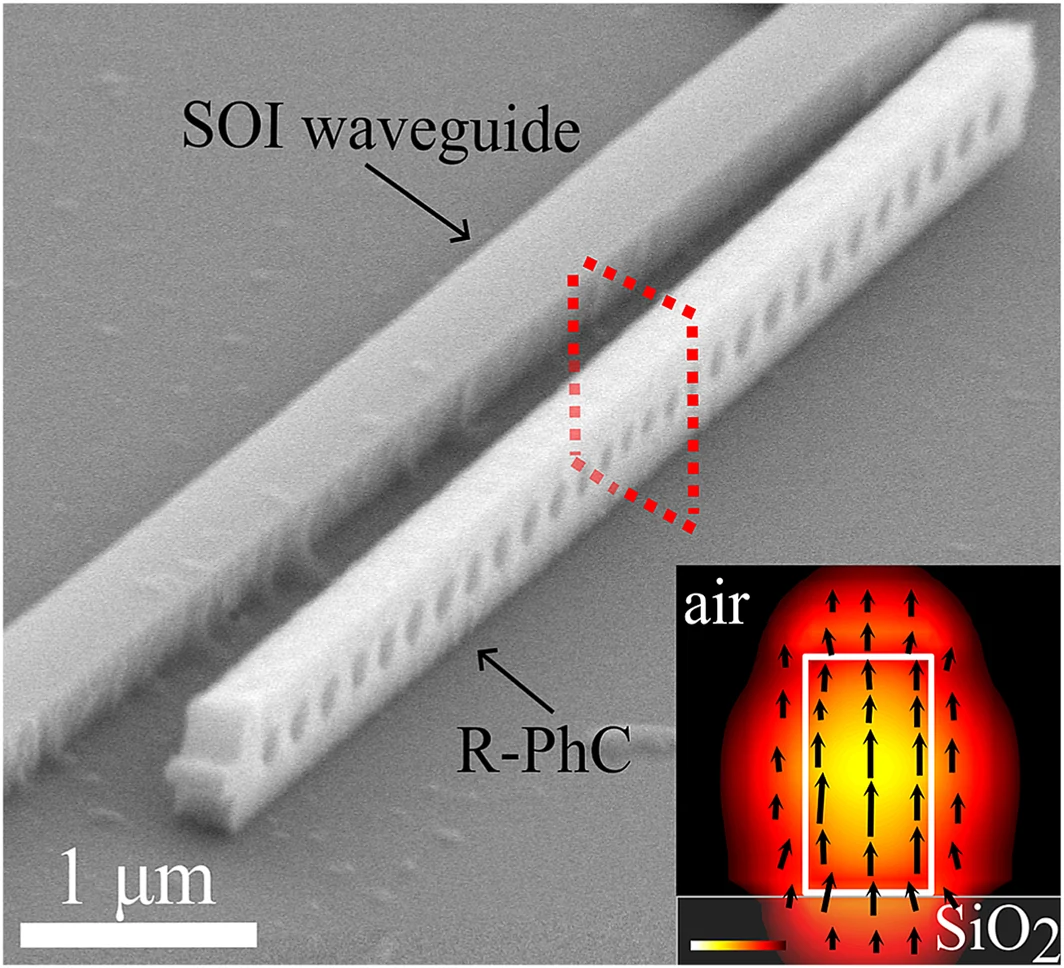

## 1 Introduction

Silicon photonics has emerged as a key platform for large‐scale optical integration, enabling high‐speed communication, signal processing, and photonic computing architectures owing to its scalability and compatibility with semiconductor manufacturing [1, 2, 3, 4, 5]. Despite these advantages, silicon inherently lacks efficient light‐generation capability, limiting the realization of active photonic functionalities [6, 7]. To overcome this fundamental constraint, heterogeneous integration has become a central strategy for extending silicon photonics beyond monolithic limits. Accordingly, substantial efforts have focused on integrating III–V compound semiconductors, such as InGaAsP, onto silicon to realize efficient on‐chip light sources [8, 9, 10]. Approaches based on direct epitaxial growth [11, 12, 13] and wafer bonding [11, 14] have demonstrated significant progress; however, they remain constrained by lattice mismatch, thermal expansion incompatibility, and stringent process requirements, which limit yield, reliability, and scalability.

In this context, transfer printing is emerging as one of the most powerful approaches for heterogeneous integration [15, 16, 17]. By enabling deterministic assembly of externally fabricated photonic devices with high spatial precision, this approach decouples material synthesis from system‐level integration, thereby mitigating thermal stress and process incompatibility. This approach has enabled the integration of a wide range of active components [18, 19, 20, 21, 22, 23, 24, 25, 26, 27, 28, 29]—including lasers [21, 22, 23, 24, 25], single‐photon emitters [26, 27], modulators [28], and photodetectors [29]—establishing transfer printing as a scalable platform for multifunctional photonic integration. Despite these advances, most transfer‐printing approaches remain fundamentally planar, where devices are integrated while preserving their original orientation. Recent efforts have begun to introduce orientation‐changing transfer schemes; for example, Katsumi et al. demonstrated a pick‐flip‐and‐place approach for deterministic inversion and integration of diamond nanobeams [30]. Nevertheless, heterogeneous integration has largely focused on positional alignment, implicitly assuming that device orientation is fixed and functionally inconsequential. This constraint limits access to orientation‐dependent optical properties—such as polarization selectivity, field symmetry, and anisotropic coupling—that are intrinsic to many nanophotonic devices [31, 32, 33]. Recent studies have demonstrated that such polarization functionalities can be engineered directly through cavity design; for example, Ryu et al. reported an intrinsically TM‐polarized photonic‐crystal (PhC) nanobeam laser [34]. However, realizing comparable polarization control through device orientation within a deterministic heterogeneous integration framework remains challenging.

In this work, we introduce a rolling‐enabled transfer‐printing strategy that enables deterministic nonplanar heterogeneous integration through precisely controlled mechanical rotation. Using this approach, identical PhC nanobeam lasers are orientation‐selectively integrated onto silicon waveguides, enabling deterministic coupling to either TE‐like or TM‐like guided modes without modifying the cavity design or material structure. We experimentally realize both planar and nonplanar configurations, demonstrating stable lasing operation and polarization‐selective emission governed by the orientation‐defined coupling mechanism. The integrated devices maintain high quality factors (Q‐factors) on the order of 10^5^ while achieving high coupling efficiencies approaching 90% in simulations. Furthermore, they experimentally demonstrate robust lasing and efficient coupling with silicon waveguides, with clearly defined TE‐ or TM‐polarized outputs depending on the integration geometry. By establishing mechanical orientation as a controllable degree of freedom in heterogeneous integration, this work extends transfer printing beyond planar placement and provides a scalable route toward polarization‐engineered, waveguide‐integrated photonic systems on silicon.

## 2 Results

To realize orientation‐controlled heterogeneous integration beyond planar architectures, we introduced a rolling‐enabled transfer‐printing strategy that enables deterministic nonplanar integration of nanolasers on silicon photonics through precisely controlled mechanical rotation. Prior to integration, the nanobeam laser exists as a free‐standing structure suspended from the substrate, hereafter referred to as the free‐standing PhC (FS‐PhC) nanobeam. As illustrated in Figure 1a,b, this approach enables two representative integration geometries—a planar configuration and a nonplanar, orthogonal configuration—both realized on a silicon‐on‐insulator (SOI) platform. Although the same nanobeam laser is used in both cases, the relative orientation between the cavity and the waveguide defines distinct optical interfaces, leading to fundamentally different coupling behaviors. In the planar transfer–integration geometry (Figure 1a), the FS‐PhC nanobeam is aligned parallel to the silicon waveguide, such that the dominant in‐plane electric‐field component of the cavity mode overlaps efficiently with the guided mode of the waveguide, favoring coupling to a TE‐like guided mode. We refer to this configuration as the planar‐integrated PhC (P‐PhC) nanobeam, which represents the canonical geometry widely adopted in transfer‐printed nanolaser integrations reported to date [23, 25]. In contrast, rotating the same FS‐PhC nanobeam by 90° and integrating it in a nonplanar, sidewall‐coupled geometry (Figure 1b) fundamentally alters the coupling symmetry. In this orientation, the out‐of‐plane electric‐field component of the nanobeam laser becomes the dominant contributor to the evanescent overlap with the waveguide, enabling selective coupling to a TM‐like guided mode without any modification to the cavity geometry, material stack, or epitaxial design. Importantly, the rolling operation does not create a distinct intrinsic TM cavity mode; rather, it physically reorients the same fundamental cavity‐mode family with respect to the silicon waveguide, thereby selecting an orthogonal guided‐polarization channel. This nonplanar, rotated configuration is hereafter referred to as the rotated PhC (R‐PhC) nanobeam. Figure 1c,d show scanning electron microscopy (SEM) images of PhC nanobeam laser devices fabricated using the rolling‐enabled transfer‐printing approach. Identical InGaAsP FS‐PhC nanobeam lasers are deterministically transfer‐printed adjacent to SOI waveguides in the P‐PhC and R‐PhC configurations, respectively, with precise control over their relative orientation. Although the detailed rolling‐enabled transfer‐printing procedure and fabrication considerations are discussed later, the SEM images shown here demonstrate the feasibility and robustness of orientation‐controlled integration at the device level. Insets displaying simulated electric‐field distributions further corroborate the orientation‐dependent coupling mechanism, revealing predominantly in‐plane field polarization for the P‐PhC cavity and out‐of‐plane polarization for the R‐PhC cavity.

**FIGURE 1:**
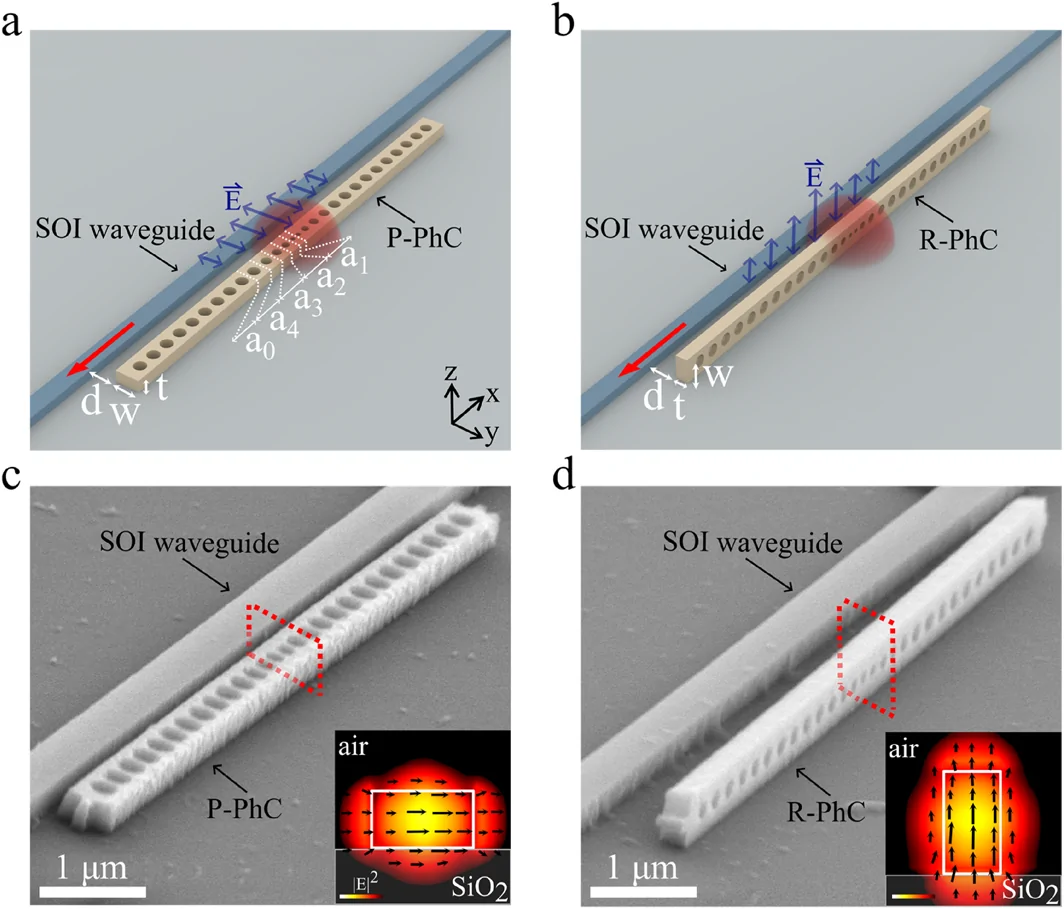
Orientation‐controlled nonplanar heterogeneous integration and waveguide coupling. (a, b) Schematics of orientation‐selective integration of an identical photonic‐crystal (PhC) nanobeam laser onto a silicon‐on‐insulator (SOI) waveguide using rolling‐enabled transfer printing: (a) Planar configuration (P‐PhC) enabling TE‐like mode coupling, and (b) nonplanar, orthogonal configuration (R‐PhC) enabling TM‐like mode coupling. The blue arrows indicate the dominant electric‐field polarization of each mode. (c, d) Scanning electron microscopy (SEM) images of the fabricated P‐PhC and R‐PhC devices, respectively, integrated in close proximity to the SOI waveguide. Insets show the corresponding simulated electric‐field intensity profiles (|*E*|^2^), illustrating the physical reorientation of the same fundamental nanobeam cavity mode and its selective coupling to the TE‐like and TM‐like guided modes of the silicon waveguide.

To quantitatively investigate the impact of orientation‐defined integration, we performed three‐dimensional finite‐difference time‐domain (3D FDTD) simulations to analyze how planar and nonplanar geometries influence the cavity characteristics—including the resonance wavelength (*λ*) and Q‐factor—as well as the optical radiation pathways and cavity–waveguide coupling behavior. Figure 2a schematically illustrates the three representative configurations: FS‐PhC, P‐PhC, and R‐PhC. The FS‐PhC cavity is based on a freestanding InGaAsP PhC nanobeam design, in which periodic air holes are patterned along the nanobeam and the lattice constant is gradually modulated along the *x*‐axis, supporting a strongly localized high‐Q resonant mode at the cavity center [23]. In this design, the nanobeam has a lattice constant (*a*_0_) of 430 nm, a thickness (*t*) of 280 nm and a width (*w*) of 580 nm, with *a*_1_ = 310 nm, *a*_2_ = 340 nm, *a*_3_ = 370 nm, and *a*_4_ = 400 nm, as defined in Figure 1a, where the air‐hole radius is given by *r*_*i*_ = 0.34 *a*_*i*_. Figure 2b compares the simulated resonance wavelengths and Q‐factors of the three configurations. Relative to the FS‐PhC cavity (*λ*_FS‐PhC_ = 1554 nm), both P‐PhC and R‐PhC configurations exhibit a redshift of the cavity resonance (*λ*_P‐PhC_ = 1570 nm and *λ*_R‐PhC_ = 1567 nm), primarily due to the increased effective refractive index introduced by the SiO_2_ substrate. The larger redshift observed for the P‐PhC configuration can be attributed to its relatively reduced nanobeam thickness (*t*) compared to its width (*w*), which enhances the modal overlap with the substrate more largely than in the R‐PhC configuration. Concurrently, both integrated configurations experience a reduction in Q‐factor (*Q*_FS‐PhC_ = 6.8 × 10^5^, *Q*_P‐PhC_ = 3.6 × 10^5^, and *Q*_R‐PhC_ = 1.4 × 10^5^) dominantly due to increased radiation loss into the substrate. The additional reduction in Q‐factor for the R‐PhC configuration arises from nonplanar integration‐induced symmetry breaking of the cavity structure along the dominant electric‐field direction, leading to a partial collapse of the photonic bandgap and enhanced radiation loss along the nanobeam (*x*) direction. Despite this degradation, both integrated geometries retain Q‐factor on the order of 10^5^, enabling stable lasing operation. These trends in Q‐factor are further corroborated by the observed redistribution of radiation pathways in Figure 2c. Upon transfer–integration onto the SiO_2_ substrate, both P‐PhC and R‐PhC cavities exhibit enhanced emission toward the substrate (−*z* direction) relative to the FS‐PhC configuration. This behavior directly reflects the reduced total internal reflection at the cavity–substrate interface, resulting in enhanced downward radiation leakage and consequently lower cavity Q‐factors. In addition, the R‐PhC configuration exhibits a higher radiation rate along the nanobeam (± *x*) direction than the P‐PhC configuration, which further confirms that the additional Q‐factor degradation of the R‐PhC case results from increased radiation loss along this direction. The mode volume remains nearly preserved, increasing only modestly from 0.235 (*λ*/*n*)^3^ for the FS‐PhC cavity to 0.278 (*λ*/*n*)^3^ and 0.275 (*λ*/*n*)^3^ for the P‐PhC and R‐PhC configurations, respectively (see Supporting Information S1: Note 1).

**FIGURE 2:**
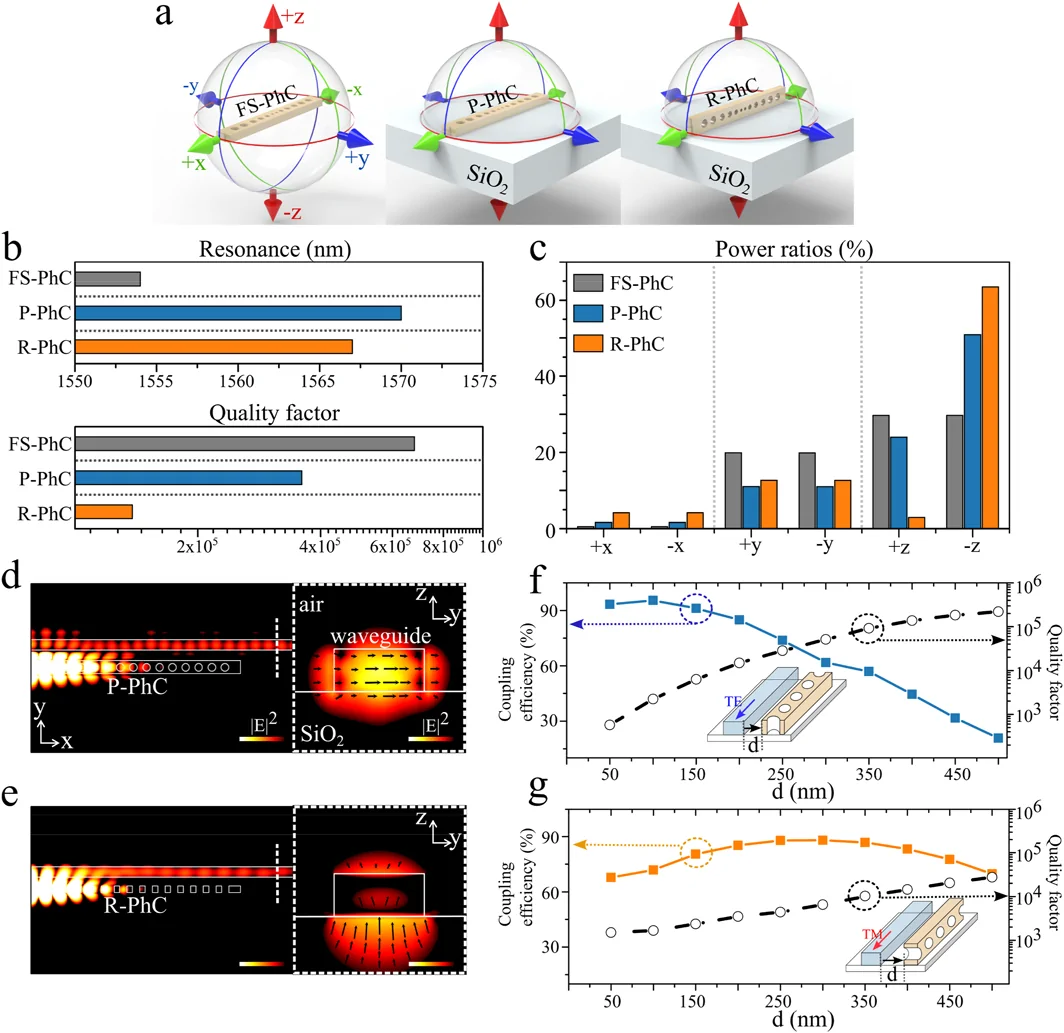
Numerical analysis of orientation‐dependent cavity properties and waveguide coupling. (a) Schematic representations of the three configurations: Free‐standing PhC (FS‐PhC), planar‐integrated PhC (P‐PhC), and rotated, nonplanar PhC (R‐PhC), illustrating their relative orientation with respect to the substrate. (b) Simulated resonance wavelengths and quality factors (*Q*) for each configuration, showing a redshift and Q‐factor reduction upon integration. (c) Directional power distribution along the ± x, ± y, and ± *z* axes, indicating the orientation‐dependent redistribution of optical radiation. (d, e) Simulated electric‐field intensity profiles (|*E*|^2^) of the P‐PhC and R‐PhC configurations, respectively, showing lateral (left) and cross‐sectional (right) field distributions and their associated polarization characteristics. (f, g) Coupling efficiency (solid lines) and coupling‐limited Q‐factor (dashed lines) as a function of edge‐to‐edge separation *d* between the cavity and the waveguide for the P‐PhC (TE‐like coupling) and R‐PhC (TM‐like coupling) configurations, corresponding predominantly to coupling into the TE‐like and TM‐like guided modes of the silicon waveguide, respectively.

We next examined the influence of planar and nonplanar configurations on evanescent coupling to an adjacent silicon waveguide. Figure 2d,e show the simulated electric‐field distributions for the P‐PhC and R‐PhC configurations, respectively, when coupled to the waveguide. The silicon waveguide is formed on a SiO_2_ substrate with a width of 500 nm and a height of 220 nm, and is designed to support both fundamental TE‐like and TM‐like guided modes (see Supporting Information S1: Note 2). In the P‐PhC geometry, the cavity predominantly couples to a guided mode whose electric‐field polarization lies parallel to the substrate, consistent with TE‐like evanescent coupling. By contrast, the R‐PhC geometry preferentially excites a guided mode with a dominant electric‐field component oriented normal to the substrate, corresponding to TM‐like coupling. These field distributions therefore demonstrate orientation‐dependent coupling of the same fundamental cavity‐mode family with orthogonal guided‐polarization channels of the silicon waveguide, rather than a transition between distinct intrinsic cavity‐mode families. Figure 2f,g present the calculated coupling efficiency and cavity Q‐factor as functions of the edge‐to‐edge separation (*d*) between the nanobeam cavity and the silicon waveguide for the P‐PhC and R‐PhC cavities, respectively. In the P‐PhC configuration (Figure 2f), efficient evanescent coupling is achieved only within a narrow separation window. The maximum coupling efficiency reaches 96% at an edge‐to‐edge distance of approximately 100 nm. At this optimal coupling point, however, the cavity Q‐factor is strongly degraded to 2.2 × 10^3^, indicating a pronounced trade‐off between coupling efficiency and cavity Q‐factor. Moreover, both the coupling efficiency and Q‐factor exhibit extremely steep variations with small changes in the cavity–waveguide separation. This behavior reflects the strong sensitivity of the coupling strength to the relative cavity–waveguide separation, as commonly observed in planar PhC–waveguide coupling schemes. In contrast, the R‐PhC configuration exhibits qualitatively different coupling characteristics, as shown in Figure 2g. The highest coupling efficiency of approximately 90% is achieved at a substantially larger separation, with an edge‐to‐edge spacing of 250 nm. Notably, at this operating point the cavity represents a relatively higher Q‐factor of 3.1 × 10^3^. More importantly, both the coupling efficiency and Q‐factor vary much more gradually as a function of separation, indicating a markedly enhanced tolerance to cavity–waveguide positioning. This behavior arises from the distinct modal‐overlap characteristics of the R‐PhC configuration, where the rotated nanobeam geometry and the more extended evanescent field of the TM‐like silicon‐waveguide mode allow appreciable cavity–waveguide overlap to be maintained over a broader separation range. As a result, the coupling strength becomes less sensitive to positional variations while still enabling efficient lateral coupling (see Supporting Information S1: Note 3).

Further insight into this distinct coupling behavior is provided by the decomposition of the cavity Q‐factor shown in Supporting Information S1: Figure S5 (see Supporting Information S1: Note 4), where the total *Q* is separated into contributions from waveguide coupling (*Q*_coupling_) and radiation/scattering losses (*Q*_rad_). For the P‐PhC configuration, *Q*_coupling_ exhibits an abrupt change within 150 nm from the nanobeam width edge, directly evidencing the highly sensitive dependence of coupling strength on separation distance. In contrast, the R‐PhC configuration shows a much more gradual variation of *Q*_coupling_ as the waveguide is displaced away from the nanobeam thickness edge, confirming its intrinsically relaxed coupling sensitivity. For both configurations, strong scattering between the cavity and the waveguide becomes dominant when the separation is reduced below 100 nm from the cavity edge, leading to a rapid decrease in *Q*_rad_. These results quantitatively demonstrate that nonplanar integration fundamentally reshapes the coupling‐efficiency–Q‐factor trade‐off, offering a more robust and fabrication‐tolerant cavity–waveguide interface compared to conventional planar coupling geometries.

To realize deterministic nonplanar heterogeneous integration of PhC nanobeam lasers, we developed a rolling‐enabled transfer‐printing process that enables controlled nanoscale rotation during device assembly, as shown in Figure 3. In conventional planar transfer printing, a µ‐PDMS stamp selectively picks up a target FS‐PhC nanobeam from a III–V wafer and transfers it onto a silicon photonic circuit while preserving its original orientation. This approach inherently fixes the geometric alignment of the device and is typically employed to achieve parallel placement for TE‐like coupling. In the present study, this conventional planar procedure was used to fabricate the P‐PhC configuration, as schematically shown in Figure 3a. To implement the nonplanar R‐PhC configuration, we introduced an additional rolling step, as depicted in Figure 3b. In this step, the detached nanobeam was mechanically rotated prior to final printing, enabling orthogonal integration of an identical device. Deterministic orientation control was achieved using two types of PDMS stamps. A larger, untreated base‐PDMS stamp (50 × 50 μm) provided mechanical support and served as a rolling platform, whereas a smaller control‐PDMS stamp (20 × 20 μm) was used to initiate and regulate rotation. The surface of the control‐PDMS stamp was treated with O_2_ plasma (50 W, O_2_ 30 sccm) to introduce a controlled adhesion contrast relative to the base stamp. By optimizing this adhesion contrast (see Supporting Information S1: Note 5), the nanobeam could undergo smooth rotational motion without being fully transferred to the control stamp, thereby enabling reproducible orientation switching prior to integration. Here, the R‐PhC‐on‐chip devices used the same nanobeam structures as the P‐PhC devices; the only difference lies in the orientation defined during the rolling step. Optical microscopy images revealed distinct color contrasts between P‐PhC and R‐PhC devices due to the opposite orientation of the photonic‐crystal hole pattern, providing immediate visual confirmation of successful rotation (see Video S1).

**FIGURE 3:**
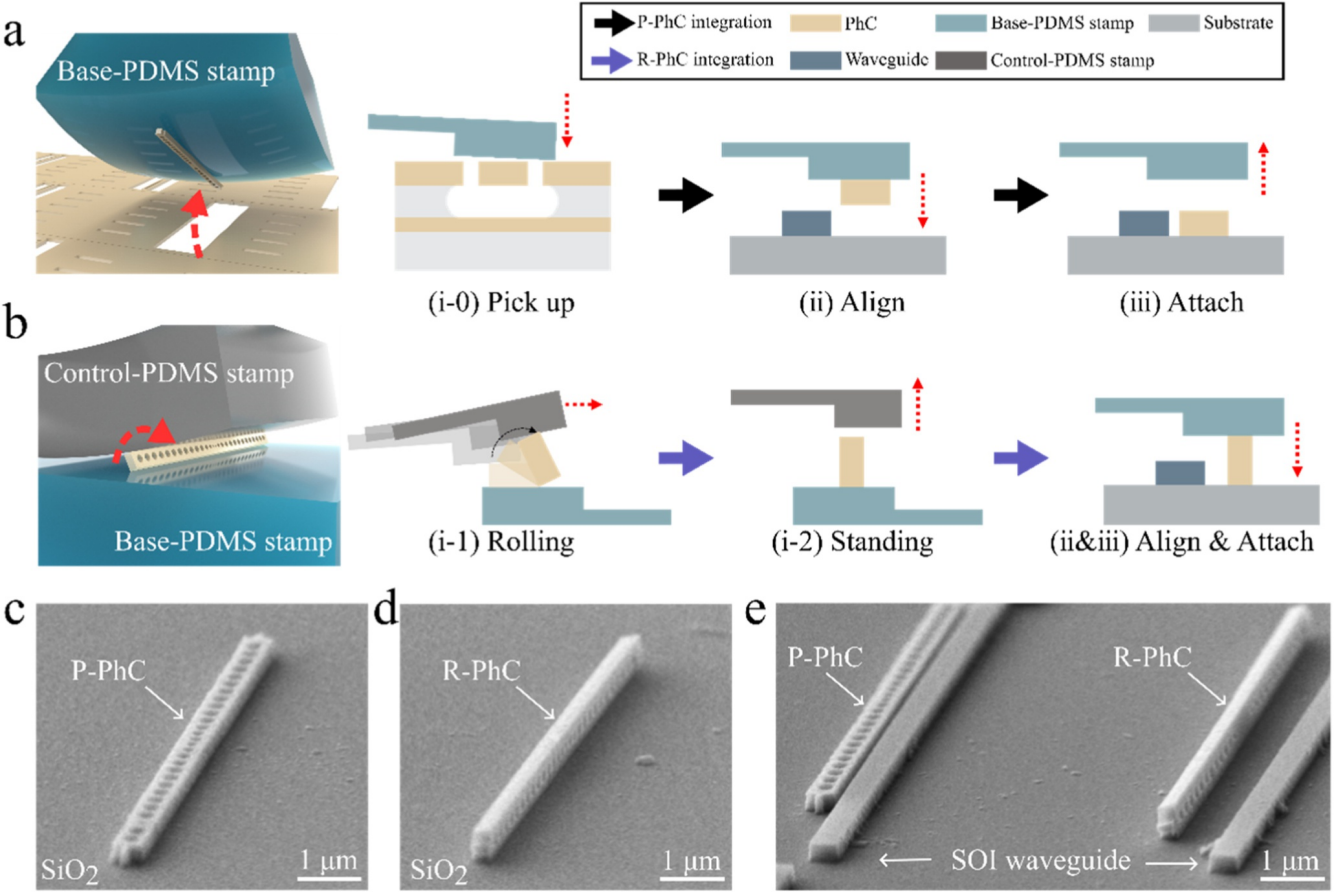
Rolling‐enabled transfer‐printing process for orientation‐controlled heterogeneous integration. (a) Pickup of a suspended photonic‐crystal nanobeam using a base‐PDMS stamp from a III–V source wafer. (b) Rolling‐enabled rotation process, in which the detached nanobeam is mechanically rotated using a control‐PDMS stamp supported by the base‐PDMS stamp, enabling deterministic orientation control. (Top right) Schematic illustrations of the transfer‐printing sequences for planar (P‐PhC) and nonplanar (R‐PhC) integration onto a silicon‐on‐insulator (SOI) platform, where both configurations share a common printing sequence, whereas the R‐PhC process includes an additional rolling step prior to final printing. (c, d) SEM images of the fabricated P‐PhC and R‐PhC devices on SiO_2_. (e) SEM image showing both P‐PhC and R‐PhC devices integrated in close proximity to an SOI waveguide.

For both planar and nonplanar integration, alignment was performed by exploiting the optical transparency of PDMS. A 50× long‐working‐distance objective lens combined with a 10× coaxial zoom objective enabled simultaneous observation of the nanobeam and the underlying silicon waveguide through the PDMS stamp. The edge‐to‐edge distance between the PhC nanobeam and the silicon waveguide was controlled at the sub‐100‐nm level. The transfer‐printing setup was mounted on a vibration‐isolated optical table and equipped with a precision tilt stage to maintain nanoscale parallel alignment during both printing and rolling processes. The InGaAsP PhC nanobeam lasers used for transfer were fabricated on an InGaAsP/InP epitaxial wafer. The active region consists of a 280‐nm‐thick InGaAsP slab containing three InGaAsP quantum wells and four barrier layers, supported by an InP sacrificial layer. One‐dimensional PhC nanobeam cavities with tapered air‐hole arrays were defined using high‐resolution electron‐beam lithography, followed by Cl_2_‐assisted accelerated Ar ion‐beam etching (CAIBE) to form smooth and vertical sidewalls. After removal of the resist mask, the InP sacrificial layer was selectively etched using an HCl/H_2_O solution to obtain free‐standing nanobeams suitable for transfer integration. SEM images in Figure 3c–e confirm that both P‐PhC and R‐PhC configurations are precisely transfer‐printed onto the SOI platform and positioned in close proximity to silicon waveguides.

To experimentally validate the orientation‐controlled heterogeneous integration approach, we characterized both the lasing behavior and the polarization‐resolved waveguide coupling of the integrated P‐PhC and R‐PhC nanobeam devices. The devices were optically pumped using a 976‐nm laser diode under pulsed excitation conditions (5% duty cycle, 50 ns pulse width, and 1 MHz repetition rate) at room temperature (see Methods and Supporting Information S1: Note 6). The pump beam was focused onto the nanobeam cavity through a 50× objective (NA = 0.65), yielding a Gaussian spot with a diameter of approximately 5 μm. The emitted radiation from the laser region and the silicon waveguide facet was collected through an infrared objective and directed to an IR CCD detector. A rotatable linear polarizer was inserted in the detection path to discriminate between TE‐like and TM‐like guided‐mode components.

With this measurement configuration, we first verified that both geometries sustain stable lasing after heterogeneous integration. Figure 4a,b show the measured emission spectra of the P‐PhC and R‐PhC nanolasers integrated on the SOI substrate. In both configurations, single dominant lasing peaks are observed above laser threshold with full width at half maximum (FWHM) values of 0.32 and 0.41 nm for the P‐PhC and R‐PhC devices, respectively, measured at a pump power of 50 μW. The corresponding light–light (L–L) characteristics (Figure 4c,d) exhibit clear threshold behavior, with absorbed pump thresholds of 38 μW for P‐PhC and 44 μW for R‐PhC. The comparable lasing behavior of the P‐PhC and R‐PhC configurations further confirms that the intrinsic cavity feedback and gain characteristics are largely preserved during mechanical reorientation. The higher threshold of the R‐PhC device is attributed to its reduced cavity Q‐factor (Figure 2b), which leads to increased optical loss. Along with clear threshold behavior, pronounced linewidth narrowing is observed near the threshold region. Below threshold, the emission linewidth remains broad, corresponding to spontaneous emission from the cavity. As the pump power approaches the threshold, the linewidth rapidly decreases to 0.38 and 0.43 nm, and subsequently stabilizes at 0.32 and 0.41 nm above threshold for the P‐PhC and R‐PhC devices, respectively. This abrupt spectral narrowing is a characteristic signature of the transition from spontaneous emission to stimulated emission, confirming coherent lasing operation in both geometries. In Figure 4c,d, the threshold values are expressed in terms of absorbed pump power, estimated using 3D FDTD absorption simulations (see Supporting Information S1: Note 7), to enable a meaningful comparison between the two configurations. The absorbed power fractions are 7.72% for P‐PhC and 6.65% for R‐PhC, reflecting slight differences in field absorption within the device. It should be noted, however, that the slopes of the measured L–L curves should not be directly compared to intrinsic laser slope efficiencies. The detected emission was collected through a monochromator slit and therefore represents only a fraction of the total emitted power. Because the P‐PhC and R‐PhC exhibit different spatial emission profiles, their collection efficiencies can differ even under the same optical setup. The observed lasing wavelength difference between the P‐PhC and R‐PhC laser devices arises from the integration‐induced resonance shift discussed in Figure 2b, together with minor fabrication‐induced variations.

**FIGURE 4:**
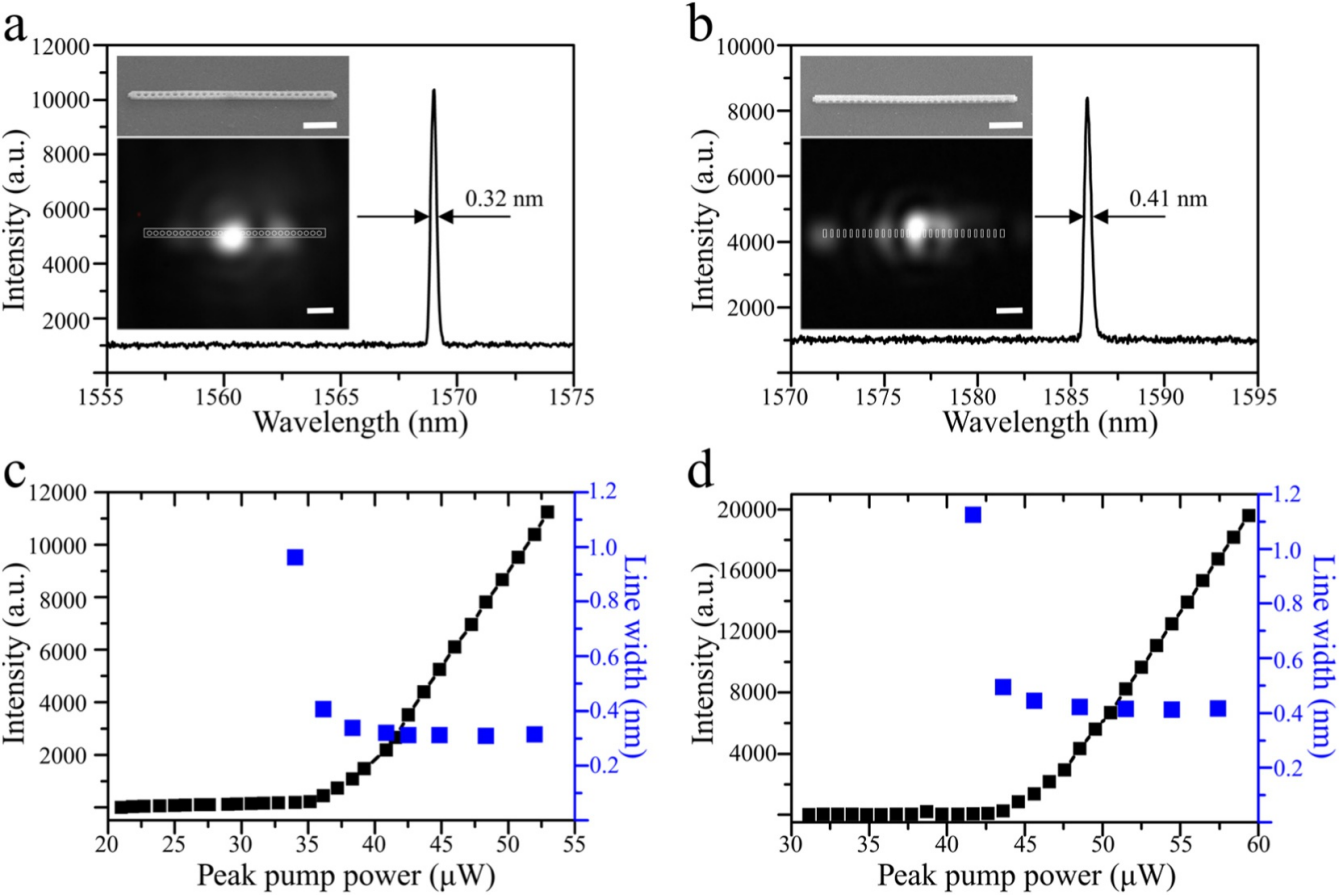
Lasing characteristics of orientation‐controlled PhC nanobeam devices. (a, b) Emission spectra of the (a) P‐PhC and (b) R‐PhC nanobeam lasers integrated on a SiO_2_ substrate, with corresponding SEM images (top insets) and emission profiles (bottom insets). The lasing peaks exhibit linewidths of 0.32 and 0.41 nm for the P‐PhC and R‐PhC devices, respectively, measured at a pump power of 50 μW. (c, d) Light–light (L–L) characteristics (black squares) and linewidth evolution (blue squares) as a function of absorbed peak pump power for the (c) P‐PhC and (d) R‐PhC devices. Both configurations show clear threshold behavior accompanied by pronounced linewidth narrowing, confirming coherent lasing operation.

We next analyzed the polarization characteristics of the waveguide‐coupled emission. Figure 5 presents polarization‐resolved measurements of P‐PhC and R‐PhC devices integrated adjacent to silicon waveguides with edge‐to‐edge separations of 135 nm (P‐PhC) and 320 nm (R‐PhC). Emission from the waveguide facet was recorded while rotating the linear polarizer through 0°–360°. For the P‐PhC device, the output intensity reaches a maximum at 10° and is suppressed at 90°, yielding a polarization extinction ratio (PER) of approximately 6.85 dB. This behavior confirms dominant TE‐like coupling, consistent with the in‐plane electric‐field orientation of the cavity mode. In contrast, the R‐PhC device exhibits maximum emission at 100° and minimum emission at 0°, corresponding to TM‐like coupling, with a measured PER of approximately 4.64 dB. The finite polarization extinction ratio can be attributed to residual vertical cavity radiation, waveguide‐facet scattering, and imperfect separation between cavity‐emitted and waveguide‐coupled components in the collection path. The angular dependence of the emitted intensity closely follows the simulated polarization response (Figure 5g,h). This experimentally observed orthogonality of the guided‐mode polarization states directly reflects the orientation‐defined symmetry of the cavity–waveguide system, rather than differences in cavity design or material structure. In addition to the guided‐mode emission from the waveguide facet, a vertically emitted component originating from the central cavity region is also observed in Figure 5c–f. This vertical radiation exhibits a polarization behavior distinct from that of the guided‐mode output, as it arises from the intrinsic cavity far‐field emission rather than waveguide‐coupled emission. As this component is not constrained by waveguide coupling conditions, its polarization state reflects the intrinsic radiation symmetry of the cavity (see Supporting Information S1: Note 8).

**FIGURE 5:**
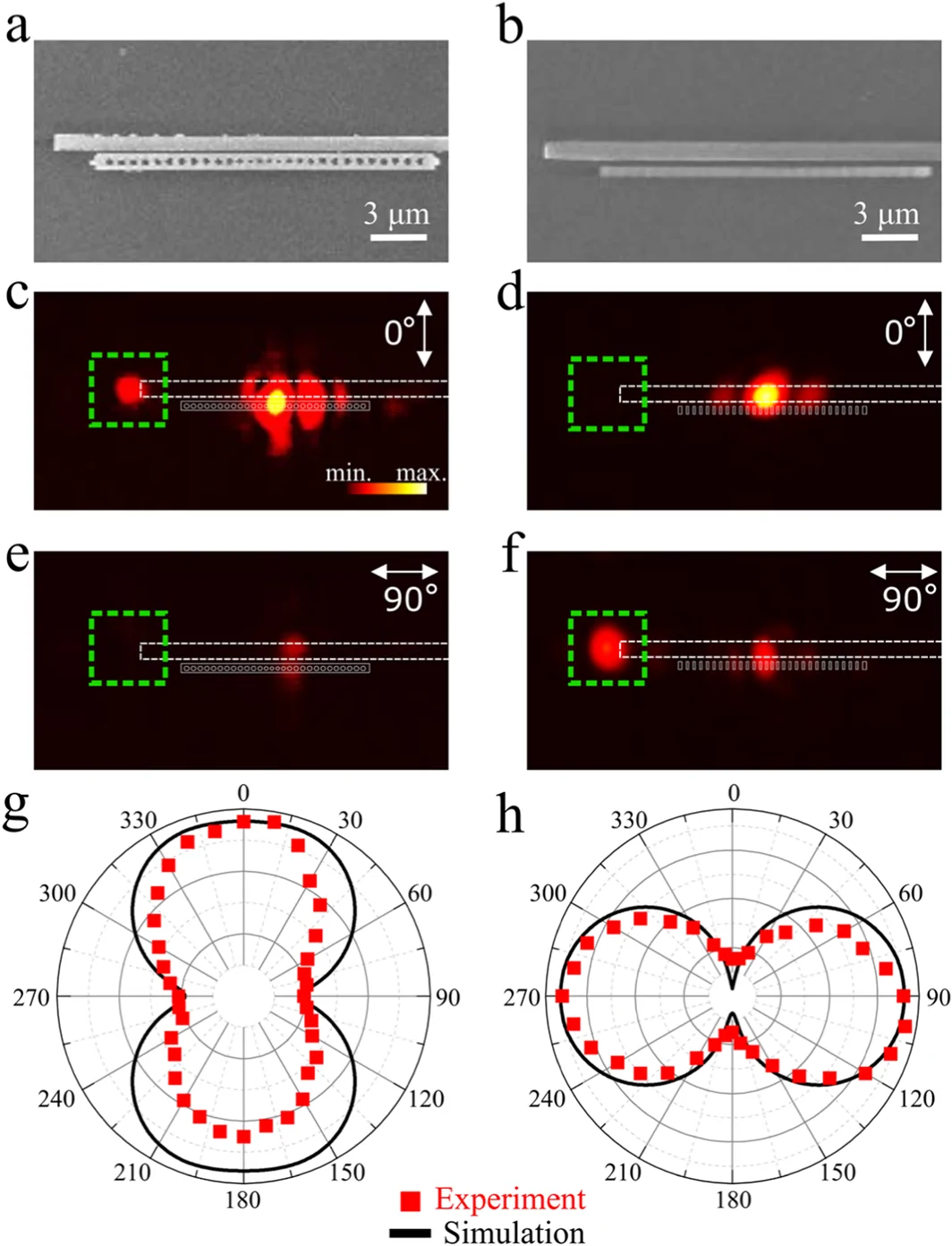
Polarization‐resolved emission and orientation‐dependent radiation characteristics. (a, b) SEM images of the (a) P‐PhC and (b) R‐PhC nanobeam devices integrated in close proximity to SOI waveguides. (c, d) Emission intensity profiles measured through a linear polarizer oriented at 0° for the (c) P‐PhC and (d) R‐PhC devices. (e, f) Emission intensity profiles measured through a linear polarizer oriented at 90° for the (e) P‐PhC and (f) R‐PhC devices. (g, h) Polar plots of the emitted intensity measured at the end facet of the SOI waveguide for the (g) P‐PhC and (h) R‐PhC configurations, comparing experimental results (red squares) with numerical simulations (black lines), highlighting orientation‐dependent polarization characteristics.

## 3 Discussion and Conclusion

We have demonstrated a rolling‐enabled transfer‐printing strategy that enables deterministic nonplanar heterogeneous integration of PhC nanobeam lasers on a silicon photonic platform. By introducing controlled mechanical rotation during transfer, identical nanobeam lasers were integrated in planar and orthogonal configurations without modifying their geometry or material composition. Both configurations exhibited stable lasing and polarization‐selective waveguide coupling, confirming that nanoscale mechanical orientation alone can define laser–waveguide coupling symmetry and polarization functionality.

Beyond the realization of polarization‐selective nanolasers, this work establishes mechanical orientation as a new and experimentally viable degree of freedom in heterogeneous photonic integration. Unlike conventional planar transfer printing, which primarily controls device position, the rolling‐based strategy enables deterministic three‐dimensional assembly at the nanoscale, thereby reshaping the trade‐off between cavity quality, coupling efficiency, and alignment tolerance. In this context, orientation is elevated from a passive geometric parameter to an active design variable for engineering optical coupling and polarization control on chip. The demonstrated approach is not limited to PhC nanobeam lasers. The ability to controllably rotate and integrate nanoscale photonic elements opens pathways toward hybrid systems such as plasmonic resonators, quantum emitters, nonlinear nanocavities, waveguide‐integrated nanophotonic devices, and metasurfaces, where anisotropic field symmetry and polarization control are critical. Although the present demonstration employs structurally simple one‐dimensional nanobeams, extension to asymmetric, irregular, or three‐dimensionally stacked structures will require further optimization of the device geometry and stamp–device contact. In particular, our experiments indicate that stable rolling is facilitated by moderate cross‐sectional aspect ratios and smooth, nearly vertical device sidewalls.

From a manufacturing perspective, the optical feedback and precision multiaxis positioning used in the present rolling process are also well suited for future automation, for example through machine‐vision‐assisted contact detection, rolling‐state tracking, and programmable orientation control. Such closed‐loop operation, together with arrayed transfer‐printing schemes, could provide a practical route toward scalable orientation‐controlled assembly. Because the III–V nanobeams are fabricated separately and subsequently integrated onto the silicon photonic platform, the process also avoids high‐temperature III–V growth or bonding directly on the finished silicon circuit, making it potentially compatible with CMOS back‐end integration. Further development toward large‐area implementation will require improved control of alignment accuracy, surface cleanliness, mechanical contact conditions, process uniformity, and array‐level yield.

More broadly, rolling‐enabled nonplanar integration opens a pathway toward three‐dimensional heterogeneous photonic architectures, enabling compact, multifunctional, and polarization‐engineered integrated photonic systems beyond the constraints of conventional planar fabrication.

## 4 Methods

### 4.1 Simulation Details

All simulations were performed using a commercial 3D finite‐difference time‐domain (FDTD) solver (Lumerical FDTD Solutions). The simulation domain was discretized with a uniform 10 nm mesh, and the refractive indices of InGaAsP, Si, and SiO_2_ were set to 3.40, 3.45, and 1.45, respectively. The photonic‐crystal nanobeam cavity was modeled with its tapered air‐hole profile in both planar (parallel) and nonplanar (orthogonal) orientations relative to the silicon waveguide. Resonant wavelengths and Q‐factors were obtained from the temporal decay of the cavity fields, and the simulated mode profiles were used to estimate the corresponding mode volumes. The coupling efficiency was evaluated by computing the ratio between the optical power flowing through a 1 μm × 1 μm monitor plane positioned at the waveguide cross‐section and the total radiated power obtained by integrating the Poynting flux over a closed surface surrounding the device.

### 4.2 Laser Fabrication

The freestanding InGaAsP photonic‐crystal (PhC) nanobeam lasers were fabricated on an epitaxial InGaAsP/InP wafer consisting of a 280‐nm‐thick InGaAsP slab containing three InGaAsP quantum wells and four barrier layers, supported by an InP sacrificial layer. To define the one‐dimensional PhC cavity and tapered air‐hole array, a 100‐nm‐thick C2 PMMA electron‐beam resist was first patterned using high‐resolution electron‐beam lithography (EBL). To employ the resist as a hard mask, we performed an irradiation process that induced cross‐linking at the resist surface, thereby enhancing its mechanical and chemical durability. Subsequently, both the InGaAsP and InP layers were dry‐etched using a Cl_2_‐assisted, accelerated Ar ion‐beam etching (CAIBE) process, where the high‐energy, short‐duration etching produced smooth and vertical sidewalls. After etching, the remaining PMMA mask was removed by O_2_ plasma ashing. Finally, the underlying InP sacrificial layer was selectively removed using an HCl/H_2_O (1:3) wet etchant, yielding a suspended PhC nanobeam structure suitable for optical characterization and transfer‐printing integration.

### 4.3 Transfer Printing and Rolling Process

Transfer printing was performed using transparent PDMS stamps to integrate FS‐PhC nanobeams onto SOI photonic chips. For planar integration (P‐PhC), a µ‐PDMS stamp was used to pick up a selected nanobeam from the III–V wafer and print it onto the silicon waveguide while preserving its original orientation. For nonplanar integration (R‐PhC), a rolling step was introduced to enable controlled mechanical rotation prior to printing. Two PDMS stamps were employed: a base‐PDMS stamp (50 × 50 μm) serving as a support platform and a smaller control‐PDMS stamp (20 × 20 μm) used to induce rotation. The control‐PDMS surface was treated with O_2_ plasma (50 W, O_2_ 30 sccm, 300 s) to create an adhesion contrast relative to the untreated base stamp. This adhesion contrast was optimized to allow smooth rotational motion without complete transfer to the control stamp (see Supporting Information S1: Note 5). Alignment was performed by exploiting the optical transparency of PDMS, enabling simultaneous visualization of the nanobeam and the underlying silicon waveguide during transfer printing. Using a 50× objective with coaxial zoom optics, a high‐precision translation stage, and the alignment scheme established in our previous transfer‐printing work [35], the target laser–waveguide edge‐to‐edge separation was controlled at the sub‐100‐nm level. The final separation was subsequently verified from posttransfer optical microscopy and SEM images. For the R‐PhC configuration, the rolling step used to rotate and stabilize the nanobeam in the upright orientation exhibited an overall success rate of approximately 70% under optimized conditions. After rotational alignment for the R‐PhC or direct alignment for the P‐PhC, the nanobeam was printed onto the SOI substrate by controlled stamp retraction. The final transfer‐printing yield was approximately 99% for the P‐PhC configuration and 95% for the R‐PhC configuration.

### 4.4 Optical Measurement Setup

The integrated P‐PhC and R‐PhC nanobeam lasers were optically pumped using a 976‐nm laser diode under pulsed excitation (5% duty cycle at 1 MHz) at room temperature. The pump beam was focused onto the nanobeam cavity through a 50× objective lens, resulting in a Gaussian spot with a diameter of approximately 5.0 μm. The incident pump power was calibrated prior to measurement using a power meter positioned at the sample plane. Emission from the cavity region and the silicon waveguide facet was collected using an infrared objective and directed to an InGaAs CCD detector coupled to a grating spectrometer with a spectral resolution of approximately 0.3 nm. For polarization‐resolved measurements, a rotatable linear polarizer was inserted in the detection path. The polarizer angle was varied from 0° to 360° in 10° steps to extract the angular dependence of the emitted intensity. To enable quantitative comparison of lasing thresholds between P‐PhC and R‐PhC devices, the absorbed pump power was estimated via full‐vector 3D FDTD absorption simulations (see Supporting Information S1: Note 7), and the measured light–light (L–L) curves were normalized accordingly. All measurements were performed under identical optical alignment and environmental conditions to ensure consistency across devices.

## Author Contributions

**Byoung Jun Park:** conceptualization, investigation, writing – original draft, writing – review and editing, visualization, methodology, validation, software, formal analysis, data curation. **Dongmin Shin:** methodology, software, data curation, formal analysis, validation, investigation, writing – original draft, writing – review and editing, visualization. **Aran Yu:** investigation, validation, formal analysis, writing – review and editing. **Byeong Uk You:** investigation, validation, data curation, methodology, software, writing – review and editing. **Jaeho Lee:** investigation, validation, writing – review and editing. **Sang‐Wook Han:** supervision, resources, funding acquisition, project administration, writing – review and editing. **Seung‐Woo Jeon:** supervision, funding acquisition, project administration, resources, writing – review and editing. **Dongyeon Daniel Kang:** supervision, writing – review and editing, resources, writing – original draft, funding acquisition, project administration, formal analysis, visualization. **Myung‐Ki Kim:** conceptualization, investigation, funding acquisition, writing – original draft, writing – review and editing, visualization, validation, methodology, software, formal analysis, project administration, resources, supervision, data curation.

## Conflicts of Interest

The authors declare no conflicts of interest.

## Supporting information


Supporting Information S1

**Video S1:** Rolling‐Enabled Transfer‐Printing Process for Controlled Rotation of a PhC Nanobeam.

## Data Availability

The data that support the findings of this study are available from the corresponding author upon reasonable request. All data generated or analyzed during this study are included in this published article and its Supporting Information files.
