# Supplementary material for: Rolling‐Enabled Nonplanar Heterogeneous Integration for Polarization‐Diverse Nanolasers on Silicon Photonics
Source: Nanophotonics. 2026 Sep 5;15(17):e70284. doi: 10.1002/nap2.70284 (PMC13672168; doi:10.1002/nap2.70284)
Supplement: Supplementary file 1 [file NAP2-15-e70284-s002.pdf]

Supporting Information

Rolling-enabled non-planar heterogeneous integration for polarization-diverse nanolasers on silicon photonics

Byoung Jun Park,† Dongmin Shin,† Aran Yu, Byeong Uk You, Jaeho Lee, Sang-Wook Han, Seung-Woo Jeon, Dongyeon Daniel Kang, and Myung-Ki Kim**

†These authors contributed equally: Byoung Jun Park, Dongmin Shin

Byoung Jun Park, Dongmin Shin, Byeong Uk You, Aran Yu, Jaeho Lee, Sang-Wook Han, Myung-Ki Kim*
KU-KIST Graduate School of Converging Science and Technology, Korea University, Seoul 02841, Republic of Korea
*E-mail: rokmk@korea.ac.kr

Byoung Jun Park
Physics & Informatics Laboratories, NTT Research, Inc., Sunnyvale, CA 94085, USA

Sang-Wook Han, Seung-Woo Jeon, Dongyeon Daniel Kang*, Myung-Ki Kim
Center for Quantum Technology, Korea Institute of Science and Technology, Seoul 02792, Republic of Korea
*E-mail: dykang@kist.re.kr

Sang-Wook Han, Dongyeon Daniel Kang
Division of Quantum Information, KIST School, Korea University of Science and Technology (UST), Seoul 02792, Republic of Korea

Table of Contents

1. Mode Volume Analysis 3

2. Mode Analysis of Silicon Waveguide 4

3. Modal Characteristics and Cavity–Waveguide Coupling 5

4. Q-Factor Decomposition and Coupling-Efficiency Optimization 7

5. PDMS Stamp Preparation and Rolling-Enabled Transfer Printing 9

6. Optical Measurement Setup 12

7. Pump Beam Absorption Calculation 13

8. Polarization Characteristics of Cavity and Waveguide Emission 14

Supplementary Video. Rolling-Enabled Transfer-Printing Process for Controlled Rotation of a PhC Nanobeam 17

Supplementary Note 1. Mode Volume Analysis

To quantitatively evaluate the effect of planar and non-planar heterogeneous integration on optical confinement, we calculated the mode volume of the PhC nanobeam cavity. The optical mode volume is defined as

$$V_{\text{mode}} = \frac{\int n^2(\mathbf{r}) \mid E(\mathbf{r}) \mid^2 \ d^3\mathbf{r}}{n^2(\mathbf{r}_{\text{max}}) \mid E(\mathbf{r}_{\text{max}}) \mid^2},$$

where $E(\mathbf{r})$ is the electric-field distribution and $n(\mathbf{r})$ is the spatial refractive index. The position $\mathbf{r}_{\text{max}}$ corresponds to the location where the electric-field energy density $n^2(\mathbf{r}) \mid E(\mathbf{r}) \mid^2$ reaches its maximum.

The mode volumes were extracted from the simulated cavity mode profiles and normalized by $(\lambda/n)^3$. The calculated values are $0.235\ (\lambda/n)^3$ for the free-standing PhC (FS-PhC) cavity, $0.278\ (\lambda/n)^3$ for the planar-integrated (P-PhC) configuration, and $0.275\ (\lambda/n)^3$ for the rotated (R-PhC) configuration, as shown in Fig. S1.

All simulations were performed under identical conditions, including a 2 μm SiO_2 substrate, a 5 nm mesh refinement in the cavity region, and perfectly matched layer (PML) boundary conditions. The presence of the substrate leads to a slight expansion of the optical mode compared to the free-standing case. Nevertheless, the P-PhC and R-PhC configurations exhibit nearly identical mode volumes on the same substrate, indicating that the orientation change introduces only a minimal perturbation to the spatial confinement of the cavity mode.

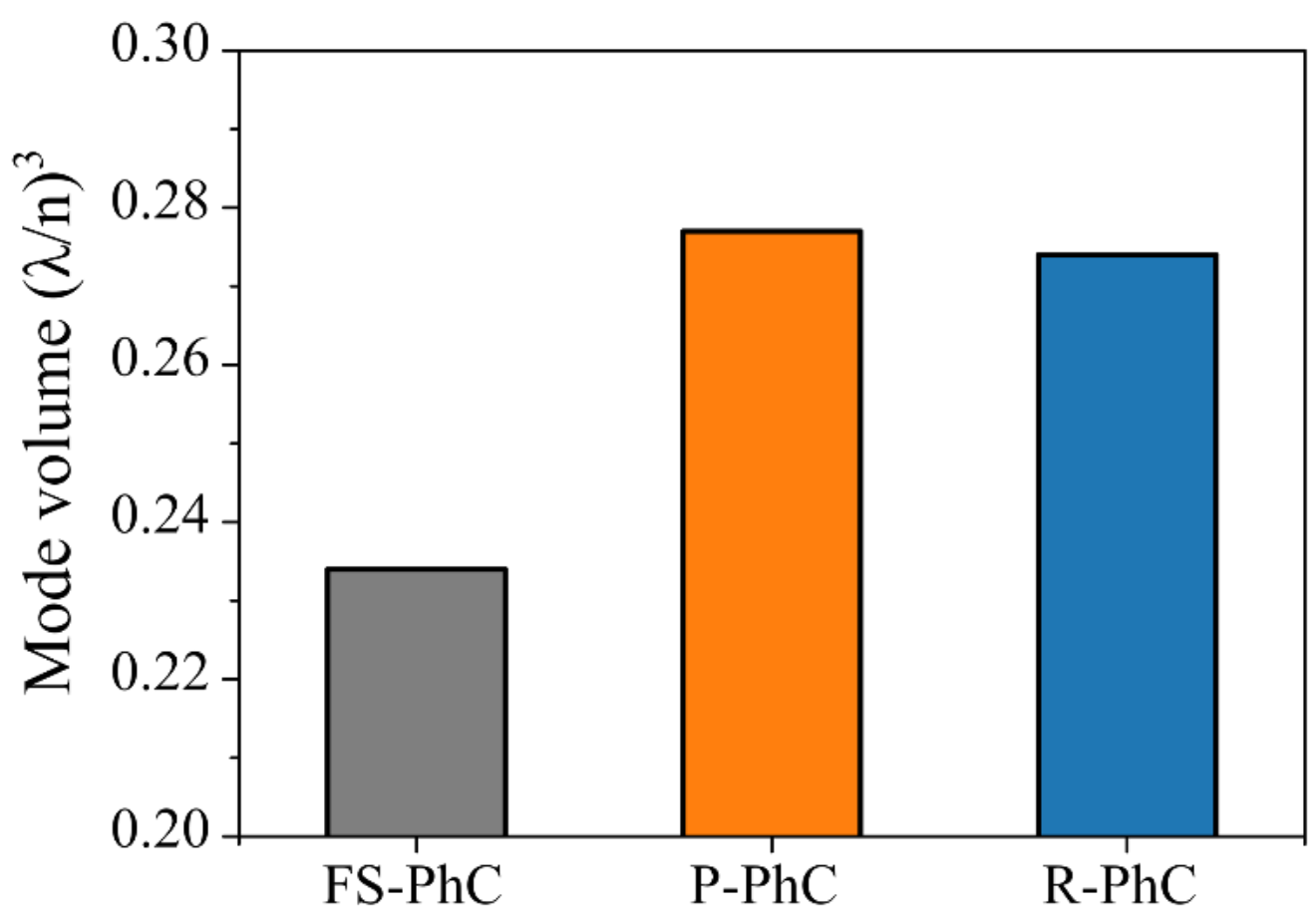

Figure S1. Mode volume comparison of the FS-PhC, P-PhC, and R-PhC configurations, normalized by $(\lambda/n)^3$.

Supplementary Note 2. Mode Analysis of Silicon Waveguide

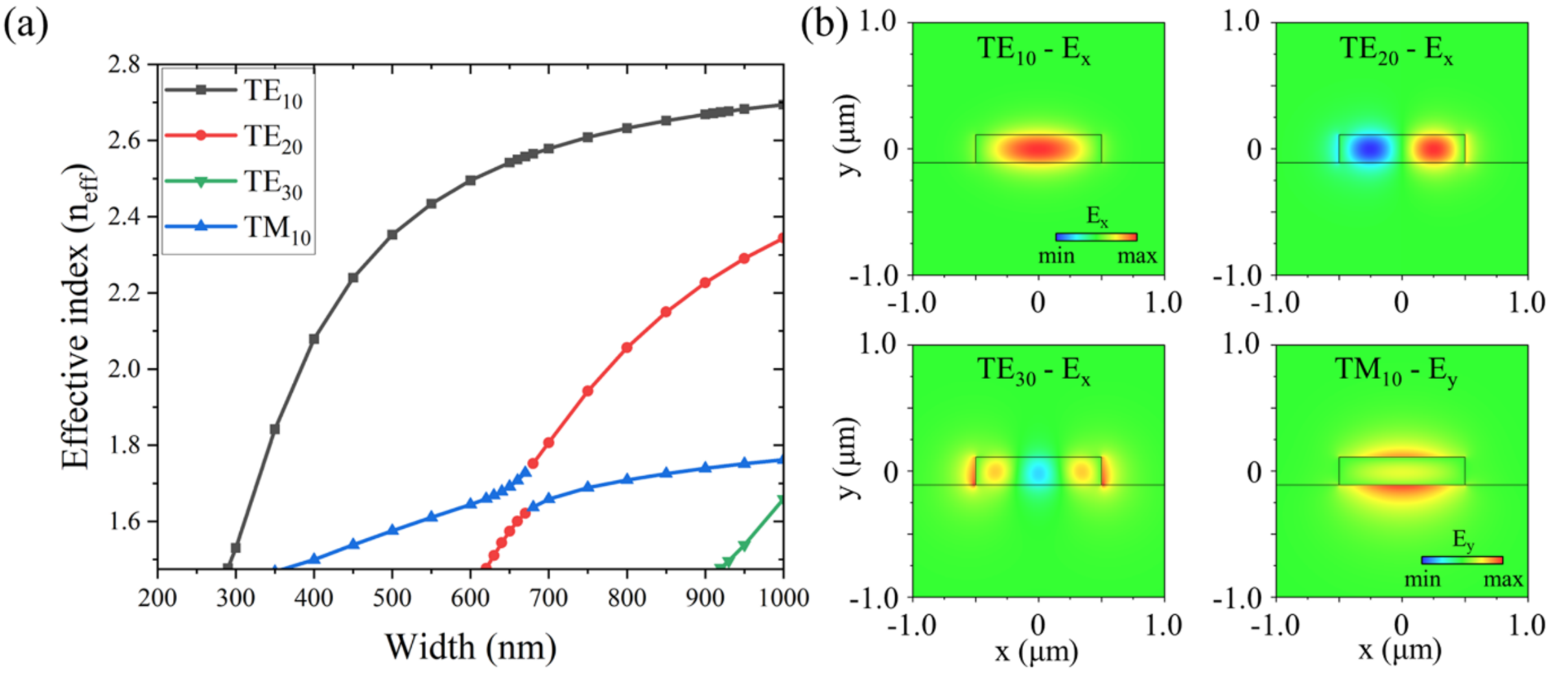

Figure S2. Mode characteristics of the silicon waveguide. (a) Calculated effective indices (n_{eff}) of guided modes as a function of waveguide width, with a fixed thickness of 220 nm. Refractive indices of 3.45 and 1.45 are assumed for silicon and silica, respectively. The dispersion curves show the evolution of TE_{10}, TE_{20}, TE_{30}, and TM_{10} modes as the waveguide width increases from 280 nm to 1000 nm. (b) Corresponding transverse electric-field profiles at a waveguide width of 1 μm, showing the dominant field components (E_x for TE-like modes and E_y for the TM-like mode). These results indicate that a waveguide width below 600 nm avoids mode crossing between the TE_{20} and TM_{10} modes, thereby minimizing excess propagation loss. Accordingly, the silicon waveguide in this work is designed with a width of 500 nm and a height of 220 nm on a SiO_2 substrate, supporting both fundamental TE-like and TM-like guided modes while suppressing higher-order mode coupling.

Supplementary Note 3. Modal Characteristics and Cavity–Waveguide Coupling

The coupling between the PhC nanobeam cavity and the silicon bus waveguide is determined by the spatial and vectorial overlap of their evanescent fields. Because the P-PhC and R-PhC configurations employ the same nanobeam cavity in different physical orientations, the principal difference in their coupling behavior originates from the relative orientation of the cavity field with respect to the silicon-waveguide modes and from the transverse confinement of those guided modes.

Figure S3 compares the normalized electric-field intensity distributions of the P-PhC and R-PhC cavity modes and the corresponding TE-like and TM-like modes of the silicon waveguide. The P-PhC and R-PhC cavity fields exhibit comparable evanescent-field extents, consistent with the fact that they originate from the same fundamental nanobeam cavity mode. Mechanical rotation of the nanobeam changes the orientation of this modal field in the laboratory frame and, consequently, the field component that overlaps with the adjacent silicon waveguide.

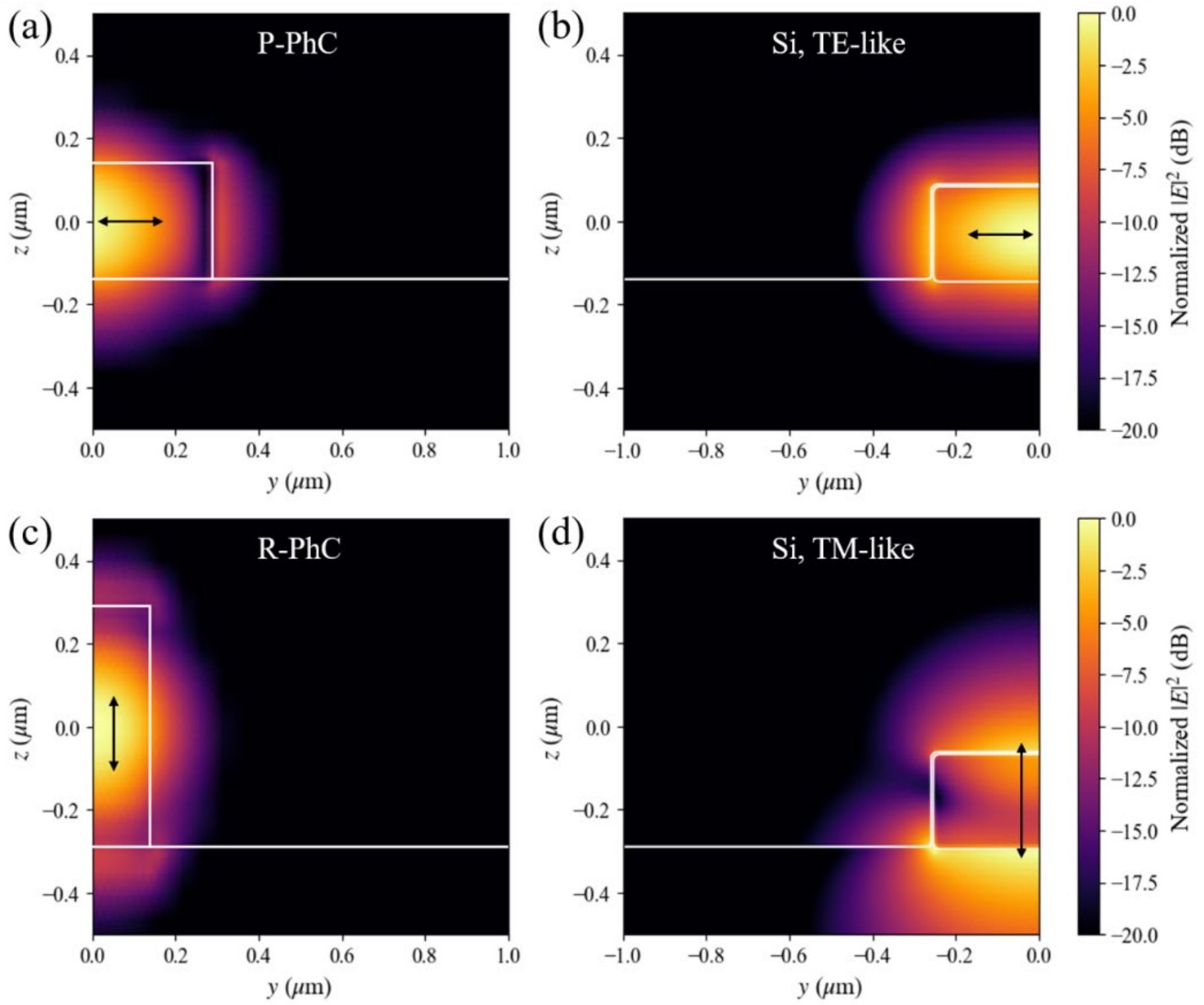

Figure S3. Modal field distributions relevant to cavity–waveguide coupling. Normalized electric-field intensity distributions of (a) the P-PhC cavity mode, (b) the TE-like guided mode of the silicon waveguide, (c) the R-PhC cavity mode, and (d) the TM-like guided mode of the silicon waveguide. The arrows indicate the dominant electric-field polarization direction in each mode. Each field distribution is independently normalized to its maximum and plotted on the same logarithmic scale. The P-PhC and R-PhC cavity modes exhibit comparable evanescent-field extents, whereas the TM-like silicon-waveguide mode shows weaker core confinement and a more extended evanescent field than the TE-like mode. This difference in transverse confinement contributes to the larger characteristic coupling distance of the R-PhC configuration.

A more pronounced difference is observed in the silicon-waveguide modes. The TM-like guided mode has a lower effective index and exhibits weaker transverse confinement than the TE-like guided mode. Consequently, the TM-like mode possesses a more extended evanescent field in the surrounding cladding, whereas the TE-like mode remains more strongly confined to the silicon core. This difference

in transverse confinement directly affects the distance dependence of the cavity–waveguide modal overlap.

The resulting coupling behavior is quantified in Figure S4 by the inverse waveguide-coupling quality factor, $1/Q_{coupling}$, as a function of the edge-to-edge cavity–waveguide separation (d). Since $1/Q_{coupling}$ is proportional to the cavity decay rate into the waveguide channel, it provides a direct measure of the cavity–waveguide coupling strength. For the P-PhC configuration, $1/Q_{coupling}$ decreases rapidly as d increases, indicating that the coupling strength is strongly dependent on the cavity–waveguide separation. In contrast, the R-PhC exhibits a much more gradual decrease of $1/Q_{coupling}$ and maintains appreciable waveguide coupling over a substantially larger separation range.

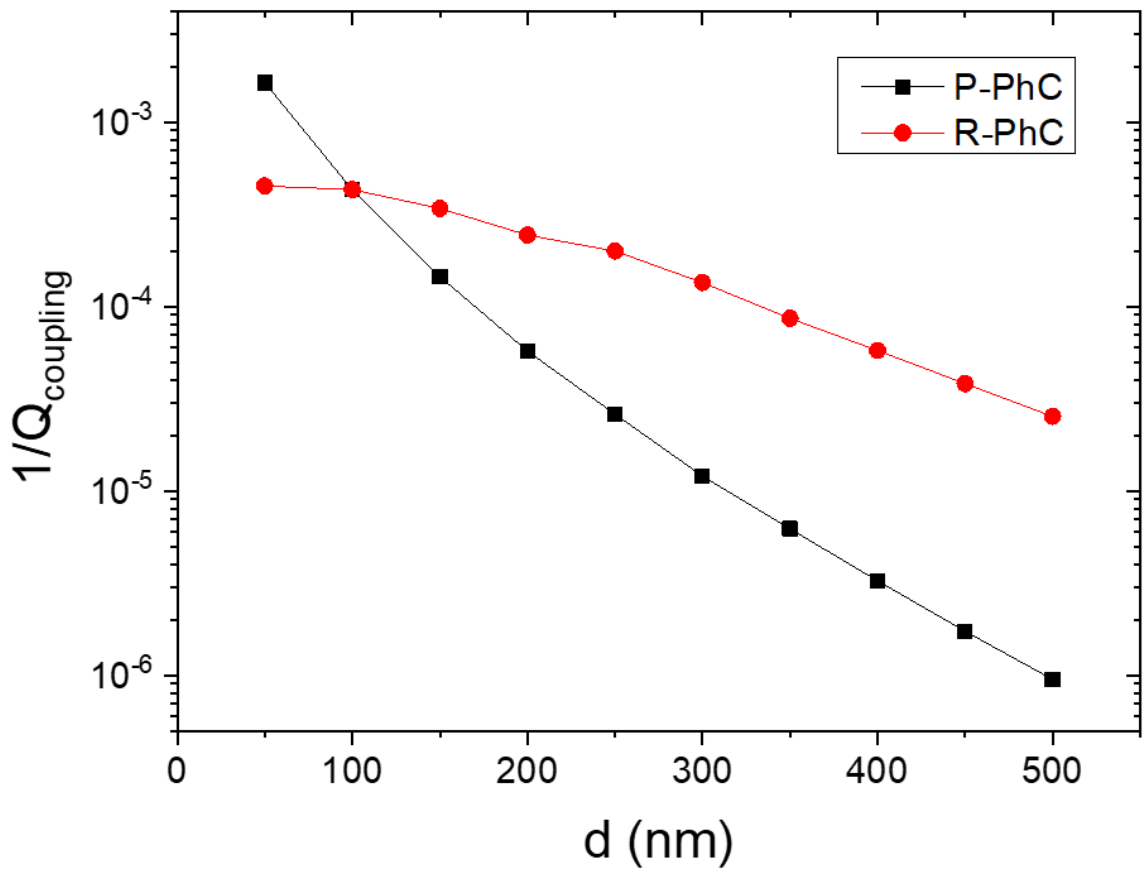

Figure S4. Cavity–waveguide coupling strength as a function of separation. Calculated inverse waveguide-coupling quality factor, $1/Q_{coupling}$, for the P-PhC and R-PhC configurations as a function of the edge-to-edge cavity–waveguide separation . The quantity represents the cavity decay rate into the waveguide channel and therefore serves as a measure of the coupling strength. The P-PhC exhibits a rapid reduction in coupling strength as increases, whereas the R-PhC maintains comparatively stronger coupling over a broader separation range. This behavior is consistent with the more extended evanescent field of the TM-like silicon-waveguide mode coupled to the R-PhC.

This difference is consistent with the modal-field distributions shown in Figure S3. Efficient coupling between the P-PhC and the more strongly confined TE-like waveguide mode requires relatively close proximity between the cavity and waveguide. In contrast, the more extended evanescent field of the TM-like waveguide mode allows the R-PhC to retain significant modal overlap at larger separations. In addition, physical rotation of the nanobeam places its thinner transverse dimension along the lateral coupling direction, further modifying the spatial relationship between the cavity and waveguide fields.

Supplementary Note 4. Q-Factor Decomposition and Coupling-Efficiency Optimization

To quantitatively analyze the optical energy decay of the cavity–waveguide system, the total cavity loss was decomposed into two contributions: waveguide coupling and radiation/scattering loss. Because material absorption was neglected in the simulations by setting the extinction coefficient to zero ($k = 0$), the total quality factor can be expressed as

$$1/\,Q_{total} = 1/Q_{coupling} + 1/Q_{rad},$$

where $1/Q_{\text{coupling}}$ represents the decay rate from the cavity into the guided mode of the silicon waveguide, and $1/Q_{\text{rad}}$ accounts for all remaining radiation and scattering losses.

The coupling efficiency is defined as the fraction of the total cavity decay that is extracted through the silicon-waveguide channel,

$$\eta = \frac{1/Q_{coupling}}{1/Q_{total}} = \frac{1/Q_{\text{coupling}}}{1/Q_{coupling}\; + \;1/Q_{rad}}.$$

Thus, the coupling efficiency is determined not only by the absolute cavity–waveguide coupling strength but also by its magnitude relative to the competing radiation and scattering channels. Consequently, a large $1/Q_{\text{coupling}}$ does not necessarily correspond to the maximum coupling efficiency if $1/Q_{\text{rad}}$ is simultaneously increased.

Figure S5 shows $1/Q_{\text{total}}$, $1/Q_{\text{coupling}}$, $1/Q_{\text{rad}}$, and the corresponding coupling efficiency as functions of the edge-to-edge cavity–waveguide separation d for the P-PhC and R-PhC configurations. For the P-PhC, $1/Q_{\text{coupling}}$ increases rapidly as the waveguide approaches the cavity, reflecting the strong distance dependence of the modal overlap. At sufficiently small separations, however, the proximity of the waveguide also strongly perturbs the cavity field and enhances scattering and radiative leakage, as evidenced by the simultaneous increase in $1/Q_{\text{rad}}$. The coupling efficiency therefore reaches a maximum at an intermediate separation of approximately d = 100 nm, where strong waveguide extraction is achieved without excessive non-waveguide loss.

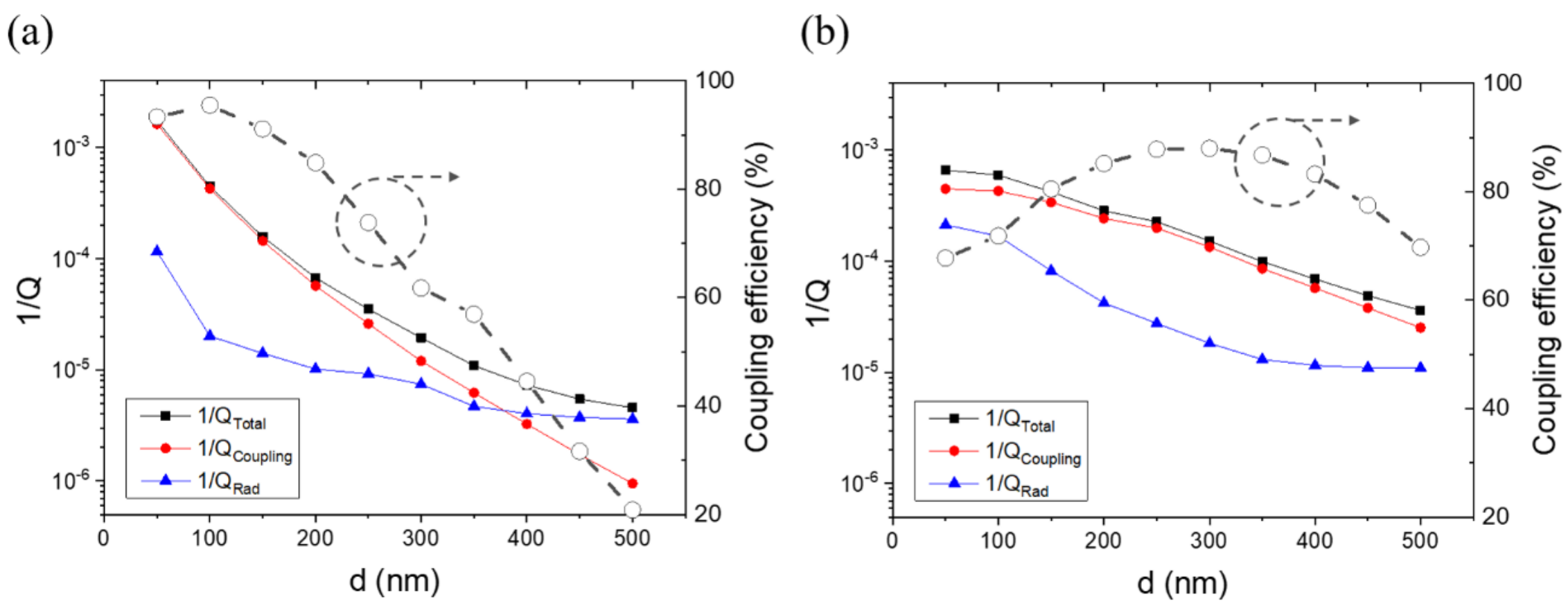

Figure S5. *Q*-factor decomposition and coupling efficiency as functions of cavity–waveguide separation. Calculated inverse quality factors and coupling efficiencies for (a) the P-PhC and (b) the R-PhC configurations as functions of the edge-to-edge cavity–waveguide separation d. $1/Q_{\text{total}}$ (black) is decomposed into the waveguide-coupling contribution $1/Q_{\text{coupling}}$ (red) and radiation/scattering contribution $1/Q_{\text{rad}}$ (blue). The coupling efficiency $\eta = (1/Q_{\text{coupling}})/(1/Q_{\text{total}})$ is plotted on the right axis. At small d, enhanced cavity–waveguide interaction increases both waveguide coupling and radiation/scattering loss, whereas at large d, waveguide coupling decreases because of reduced modal overlap. The balance between these decay channels results in maximum coupling efficiencies near $d \approx 100$ nm for the P-PhC and $d \approx 250$ nm for the R-PhC.

The R-PhC exhibits a qualitatively different distance dependence. As discussed in Supplementary Note 3, the more extended evanescent field of the TM-like silicon-waveguide mode allows appreciable cavity–waveguide coupling to persist over a substantially larger separation range. Accordingly, $1/Q_{\mathrm{coupling}}$ decreases more gradually with increasing d than in the P-PhC configuration. At very small separations, $1/Q_{\mathrm{rad}}$ again increases because of the enhanced perturbation and scattering introduced by the nearby waveguide. As a result, the maximum coupling efficiency occurs at a larger separation, near d = 250 nm, where waveguide-mediated decay remains strong while radiation/scattering loss is comparatively reduced.

These results demonstrate that the optimum coupling distance is determined by the competition between waveguide-mediated cavity decay and non-waveguide radiation/scattering loss. The distinct optimum separations and distance dependences of the P-PhC and R-PhC therefore arise from their different cavity–waveguide coupling strengths and their corresponding responses to waveguide-induced perturbation. In particular, the broader $1/Q_{\mathrm{coupling}}$ dependence of the R-PhC allows high coupling efficiency to be maintained over a wider range of cavity–waveguide separations.

Supplementary Note 5. PDMS Stamp Preparation and Rolling-Enabled Transfer Printing

Reproducible fabrication of PDMS stamps with well-defined geometry and flat contact surfaces is essential for achieving stable contact and controlled rolling during transfer printing. To fabricate the structured stamps, Si master molds with lateral dimensions of 20 × 20 μm² and 50 × 50 μm² were prepared by photolithography followed by deep reactive-ion etching to a depth of approximately 30 μm (Figure S6a). The molds were subsequently coated with Teflon to facilitate clean release of the cured PDMS while preserving a flat and defect-free contact surface. The PDMS base and curing agent were mixed at a weight ratio of 10:1 and poured onto the Si master molds after vacuum degassing (Figure S6b). The PDMS was pre-cured at 60 °C for 50 min and subsequently cured under ambient conditions for more than 24 h. The cured PDMS was then carefully released from the molds. The resulting 20 × 20 μm² and 50 × 50 μm² structures were used as the control-PDMS and base-PDMS stamps, respectively. Importantly, both stamps were fabricated using the same PDMS composition and curing conditions. We therefore avoided intentionally modifying their bulk mechanical properties, such as elastic modulus and viscoelastic response, during adhesion optimization.

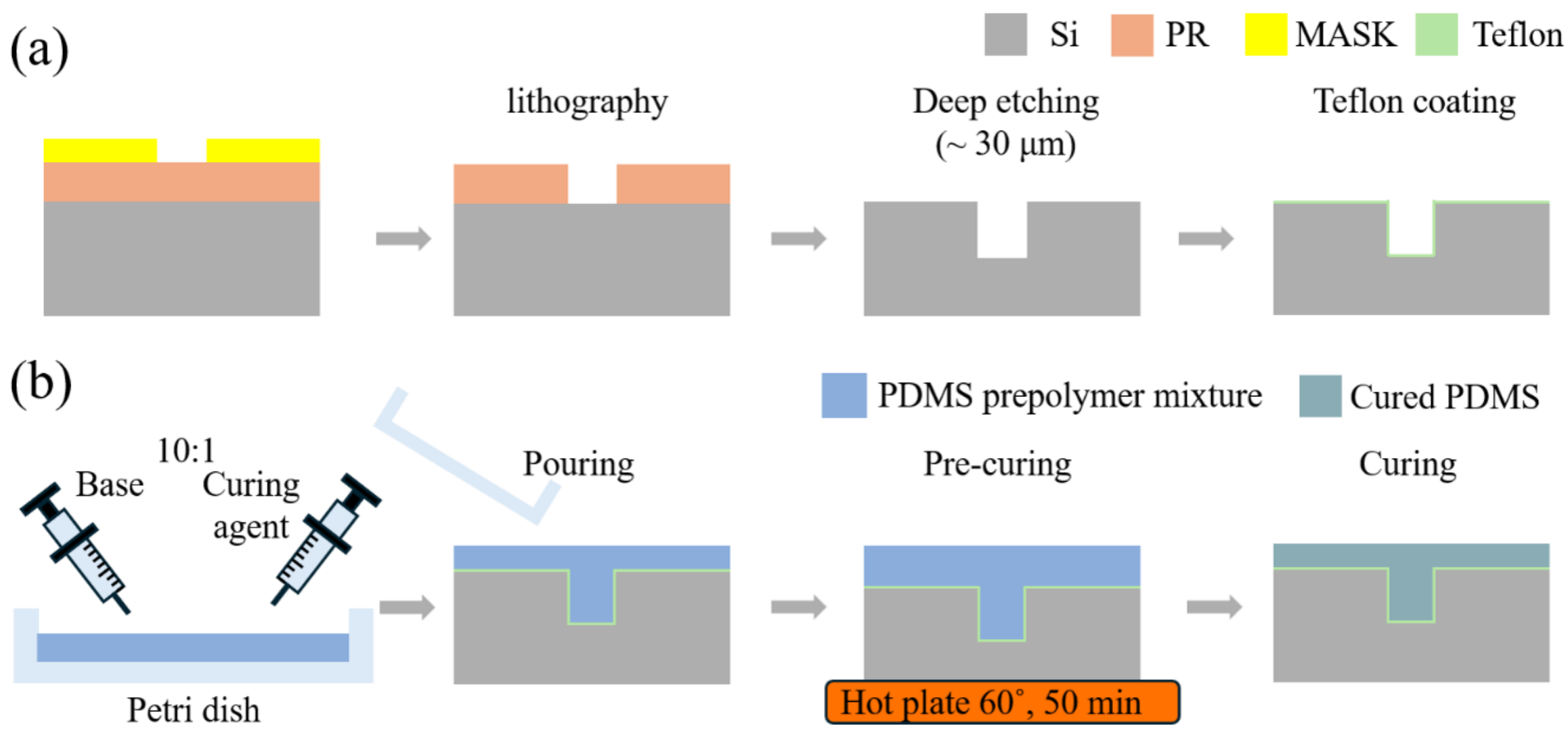

Figure S6. Fabrication process of the structured PDMS stamp. (a) Fabrication of Si master molds by photolithography and deep reactive-ion etching to a depth of approximately 30 μm, followed by Teflon coating to facilitate clean release of the cured PDMS. (b) Preparation of the PDMS stamps using a 10:1 weight ratio of PDMS base to curing agent. The degassed prepolymer mixture was poured onto the Si molds, pre-cured at 60 °C for 50 min, and subsequently cured under ambient conditions for more than 24 h. The fully cured PDMS was then carefully released from the molds to obtain the structured control- and base-PDMS stamps.

Preliminary rolling experiments showed that controlled nanobeam rotation was difficult when the control- and base-PDMS stamps had similar surface conditions. Under these conditions, the adhesion contrast between the two interfaces was insufficient to reliably initiate and sustain rolling of the PhC nanobeam. Rather than changing the PDMS base-to-curing-agent ratio, which would simultaneously modify its bulk elastic and viscoelastic properties, we selectively engineered the surface condition of the cured PDMS stamps using O_2-plasma treatment. O_2-plasma treatment oxidizes the near-surface region of PDMS and modifies its surface chemistry and interfacial adhesion through the formation of oxidized silicon-containing surface groups [S1]. In the present process, this treatment was therefore used primarily to generate an adhesion contrast between the control- and base-PDMS stamps while maintaining the same nominal bulk PDMS composition. Both stamps were treated using the same plasma power and gas-flow conditions (50 W and O_2 30 sccm), while the exposure duration was varied independently.

As summarized in Figure S7b, successful rolling was preferentially observed for selected combinations in which the control-PDMS stamp received a longer plasma treatment than the base-PDMS stamp. This result indicates that an appropriate difference in interfacial adhesion, rather than a change in bulk PDMS composition, is a key factor governing controlled nanobeam rotation. The symbols in Figure S7b summarize the qualitative outcomes of preliminary repeated rolling tests. "O" denotes a condition under which at least one successful rolling event was observed, whereas "X" denotes a condition for which successful rolling was not observed during the conducted trials. Because the number of trials was not systematically fixed for all plasma-treatment combinations, these data should be regarded as an empirical screening of the usable adhesion conditions rather than a quantitative measurement of rolling yield. Furthermore, the surface energy, work of adhesion, elastic modulus, and viscoelastic response of the PDMS stamps were not independently measured. We therefore do not assign the observed rolling behavior to a quantitatively determined change in any single material property, but rather to the overall plasma-induced modification of the PDMS surface condition and interfacial adhesion.

(a)

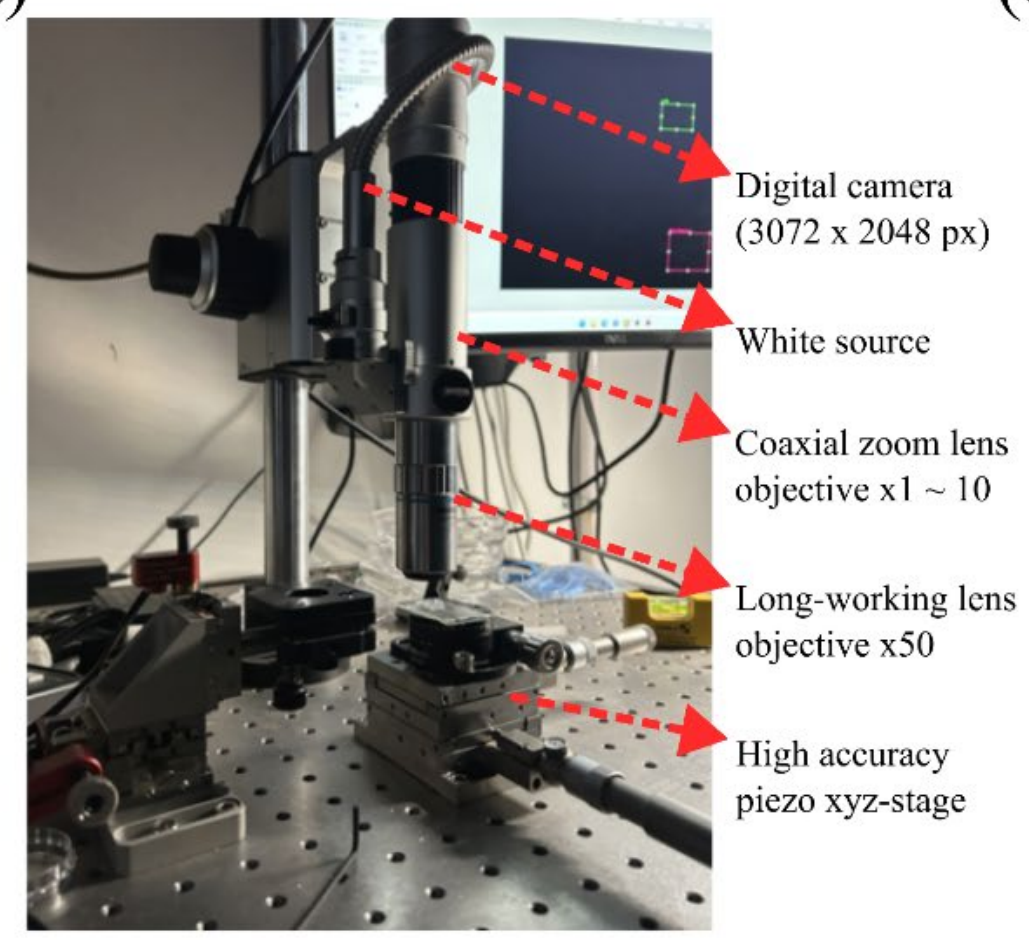

(b)

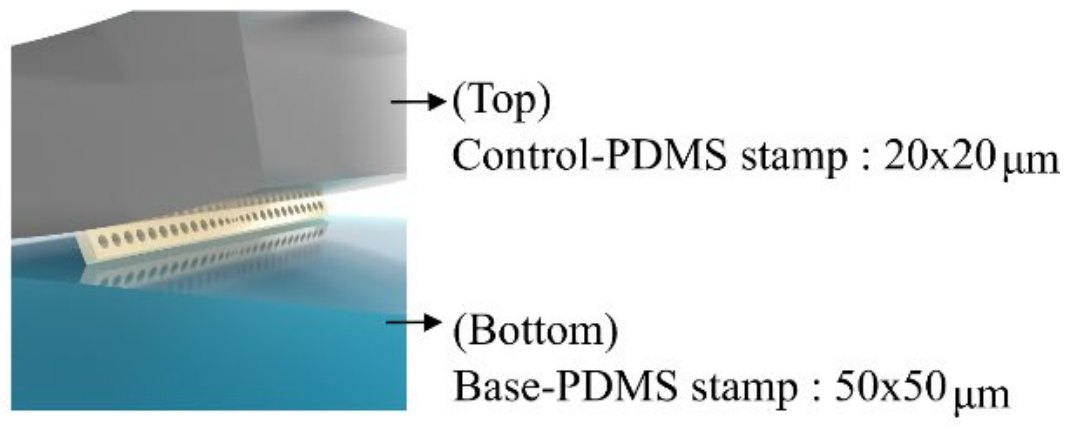

Bottom \ Top	0 min	2 min	4 min	6 min
0 min	x	x	o	o
2 min	x	x	x	o
4 min	x	x	x	x
6 min	x	x	x	x

Figure S7. Experimental setup and adhesion engineering for rolling-enabled transfer printing. (a) Optical transfer-printing setup used for the rolling process. The system integrates a PDMS stamp with an optical microscope, a long-working-distance objective, a digital camera, and a high-precision piezoelectric x–y–z stage. This configuration enables synchronized multi-axis motion during the transfer process, allowing controlled rolling of the suspended PhC nanobeam. The rotation is monitored in real time through the optical imaging system, where a distinct contrast change in the nanobeam provides direct visual confirmation of successful rotation. (b) Optimization of PDMS adhesion conditions for deterministic orientation control. Both the base-PDMS and control-PDMS stamps are treated under identical O_2 plasma conditions (50 W, 30 sccm), while the exposure time is selectively varied to tune the adhesion contrast between the two stamps. This controlled adhesion contrast enables smooth rotational motion of the nanobeam without unintended transfer to the control stamp, thereby allowing reproducible orientation switching during the rolling process. Although adhesion contrast is the dominant parameter governing the rolling transfer, external factors such as humidity, temperature, and nanobeam sidewall roughness can also influence the transfer behavior.

The rolling operation was performed using the optical transfer-printing setup shown in Figure S7a, which integrates transparent PDMS stamps with a long-working-distance objective, a digital camera, and a high-precision piezoelectric x–y–z stage. The transparency of PDMS enabled real-time observation of the contact state during rolling [see Supplementary Video]. The contacted region appeared darker in the optical-microscope image, while the separating side became brighter during rotation. This contrast provided visual feedback for controlling the lateral and vertical motion of the control stamp and for terminating the process after the desired orientation was reached. In the present

setup, contact pressure and rolling speed were not independently force- or velocity-controlled, and no active thermal control was employed during rolling. Instead, the process was regulated through incremental piezoelectric-stage motion combined with real-time optical feedback. Under the optimized adhesion conditions, the rolling process exhibited an overall success rate of approximately 70%, with successful events defined by controlled rotation to the intended orientation without visible structural damage or unintended transfer to the control stamp. These results identify interfacial adhesion contrast and real-time contact control as the key practical parameters governing reproducible rolling in the present system.

The present rolling process is performed sequentially for individual nanobeams and therefore has not yet been optimized for high-throughput manufacturing. The major time-consuming steps are optical alignment, controlled rolling, and verification of the final device orientation. These steps are currently assisted by real-time optical imaging and precision stage control, and their automation through machine-vision-based feedback and programmable stage operation would be expected to substantially improve the fabrication throughput.

[S1] Chau, K., Millare, B., Lin, A. *et al.* Dependence of the quality of adhesion between poly(dimethylsiloxane) and glass surfaces on the composition of the oxidizing plasma. *Microfluid Nanofluid* **10**, 907–917 (2011).

Supplementary Note 6. Optical Measurement Setup

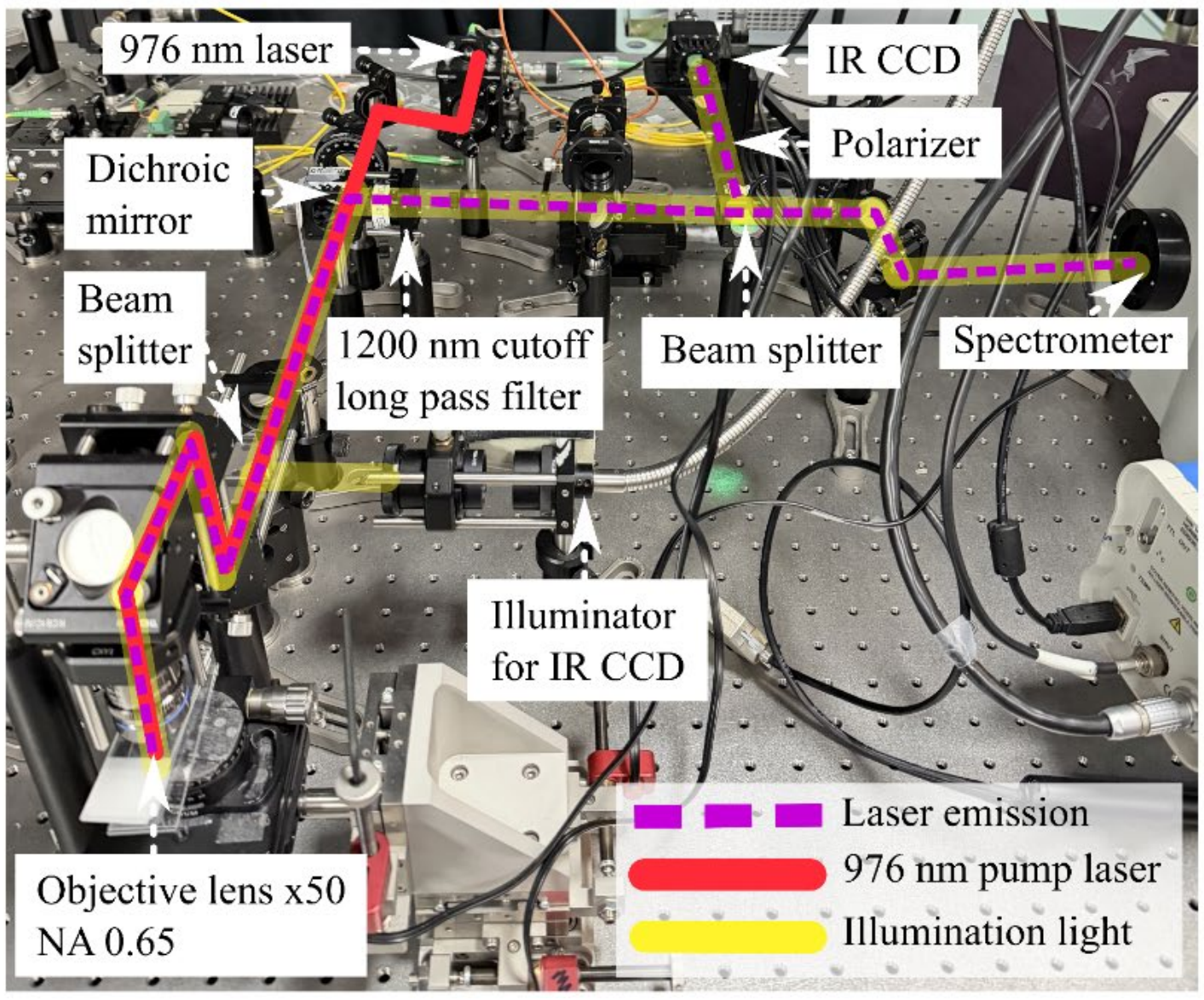

Figure S8. Optical measurement setup.

The optical setup is designed to enable pump excitation and emission characterization of the PhC nanobeam lasers, as illustrated in Fig. S8. The optical paths are distinguished by color-coded dashed lines, where the emission signal, pump beam, and illumination light are indicated separately for clarity.

A 976 nm laser diode is used as the excitation source and is directed to the device through a dichroic mirror. The pump beam is focused onto the PhC nanobeam using a 50× objective lens (NA = 0.65). The emission from the nanobeam cavity is collected through the same objective in an epi-collection configuration and is spectrally separated from the pump beam using the dichroic mirror together with a 1200 nm long-pass filter.

The collected emission is then divided into two optical paths using a beam splitter. One path is directed to an IR CCD camera for real-time imaging of the emission profiles, while the other is coupled to a spectrometer for spectral analysis, including linewidth and threshold characterization.

A separate illumination source is used to facilitate device alignment and positioning. This illumination is turned off during optical measurements to avoid background interference. For polarization-resolved characterization, a linear polarizer is placed in front of the IR CCD, and the emission intensity is recorded as a function of the polarizer angle.

Supplementary Note 7. Pump Beam Absorption Calculation

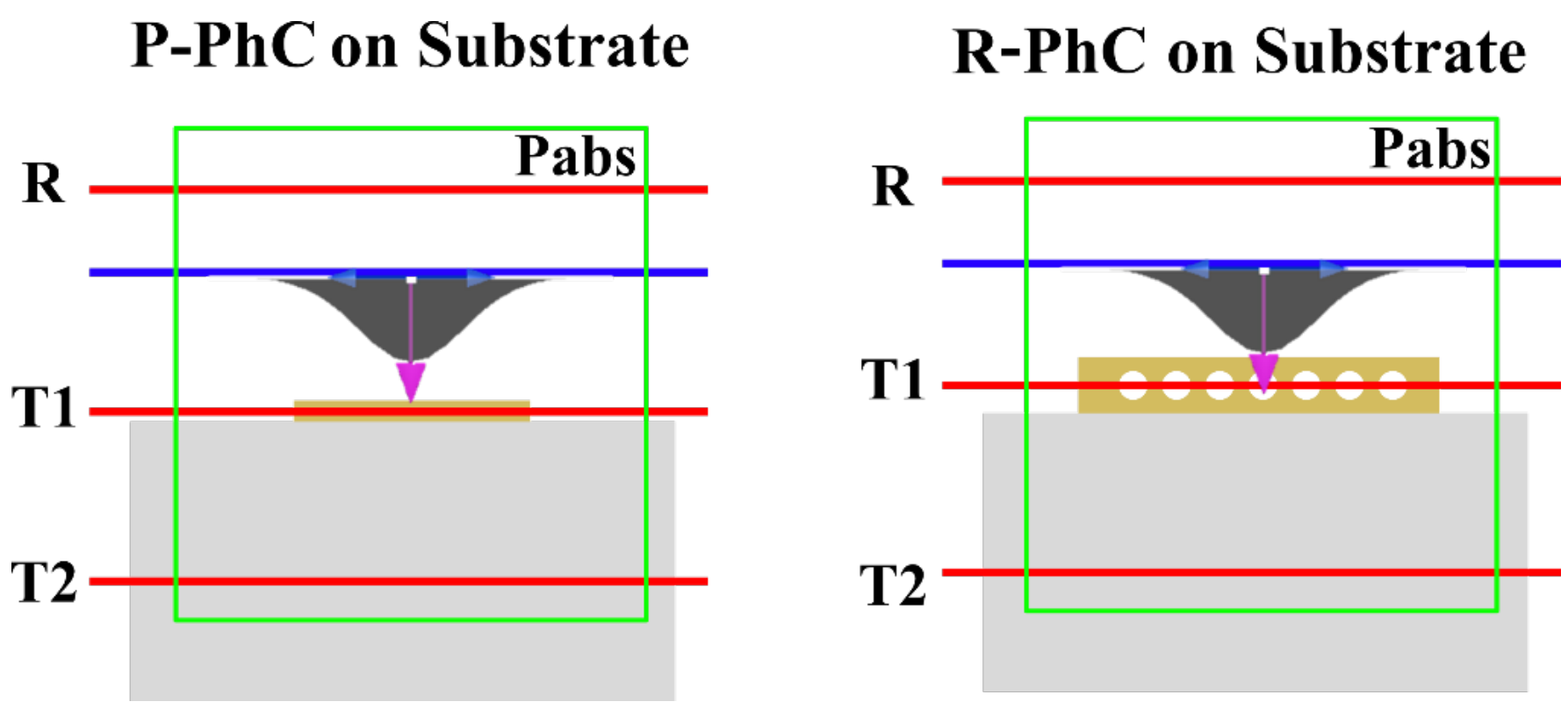

Figure S9. | 3D FDTD model used to estimate pump absorption in the integrated nanobeam structures.

To accurately evaluate the absorbed pump power in the PhC nanobeam cavities, we calculated the spatial pump-beam distribution and absorption profile using a full-vector three-dimensional finite-difference time-domain (3D FDTD) simulation.

A 976 nm Gaussian excitation beam is applied to the integrated PhC nanobeam structure, and the local absorbed power density is given by

$$P_{\text{abs}} = \frac{1}{2}\,\omega\,|\,E\,|^2\;\text{Im}(\varepsilon),$$

where $|\,E\,|^2$denotes the electric-field intensity and $\text{Im}(\varepsilon)$represents the material loss component. The volumetric absorption profile is integrated over the active cavity region to obtain the total absorbed pump power.

For the P-PhC configuration on the SiO_2 substrate, the absorbed power fraction is calculated to be 7.72%, with reflection $R = 6.31\%$ and transmission components $T_1 = 90.10\%$ and $T_2 = 85.96\%$. For the R-PhC configuration, the absorbed power fraction is 6.65%, with $R = 4.32\%$, $T_1 = 91.60\%$, and $T_2 = 89.13\%$. These differences reflect slight variations in field distribution and absorption within the cavity depending on the integration geometry.

The calculated absorption fractions are used to normalize the measured light–light (L–L) characteristics, ensuring that the comparison between P-PhC and R-PhC devices reflects intrinsic cavity properties.

Supplementary Note 8. Polarization Characteristics of Cavity and Waveguide Emission

The polarization characteristics of the emitted light were analyzed by separating two distinct radiation channels: vertical emission from the PhC nanobeam cavity and vertically scattered emission from the end facet of the silicon waveguide. These two emission channels originate from different physical processes and therefore should be interpreted separately.

Figure S10 compares the cavity-mode distributions and vertically emitted polarization characteristics of the P-PhC and R-PhC configurations. The fabricated structures are shown in Figures S10(a) and S10(b), while Figures S10(c) and S10(d) present the calculated electric-field intensity distributions $|E|^2$ in the central vertical plane of the cavities. Although the same fundamental nanobeam cavity mode is physically reoriented by 90° in the R-PhC configuration, the vertically emitted polarization is governed by the symmetry of the radiating field components rather than by a direct mapping of the dominant intracavity-field orientation.

To clarify this point, Figures S10(e–h) show the real parts of the E_x and E_y components tend to cancel, while the even-symmetric E_y components add constructively. Consequently, the vertically emitted cavity radiation is predominantly y-polarized for both cavity orientations. This symmetry-based interpretation is consistent with the polarization-resolved measurements shown in Figures S10(i) and S10(j). The measured polarization responses agree well with the calculated results and confirm that both P-PhC and R-PhC exhibit predominantly y-polarized vertical cavity emission. Therefore, the vertical polarization of the R-PhC should not be interpreted as evidence of a polarization conversion from a TM-like cavity mode. Rather, it arises from the mirror symmetry of the radiating field components of the physically rotated fundamental cavity mode.

The polarization observed at the silicon-waveguide output originates from a different radiation channel. To isolate this contribution, we separately simulated the vertical radiation generated when the fundamental TE-like and TM-like guided modes propagate to the termination of the silicon waveguide, as shown in Figure S11(a). The field distributions above the waveguide end are presented in Figures S11(b) and S11(c), and the corresponding polarization responses are shown in Figures S11(d) and S11(e).

For the TE-like guided mode, the vertically scattered emission is dominated by the y-polarized transverse field component. In contrast, the TM-like guided mode produces predominantly x-polarized vertical radiation at the waveguide termination. This difference reflects the distinct vectorial field distributions of the two guided modes and their scattering into vertically propagating radiation at the waveguide end.

These results establish a clear distinction between the two experimentally observed polarization channels. The direct vertical emission from both P-PhC and R-PhC cavities is predominantly y-polarized because of the mirror symmetry of the cavity radiation, whereas the waveguide-end emission preserves the polarization distinction between the TE-like and TM-like guided modes, yielding predominantly y- and x-polarized vertical radiation, respectively. This distinction provides the physical basis for interpreting the polarization-resolved measurements of the cavity and waveguide outputs.

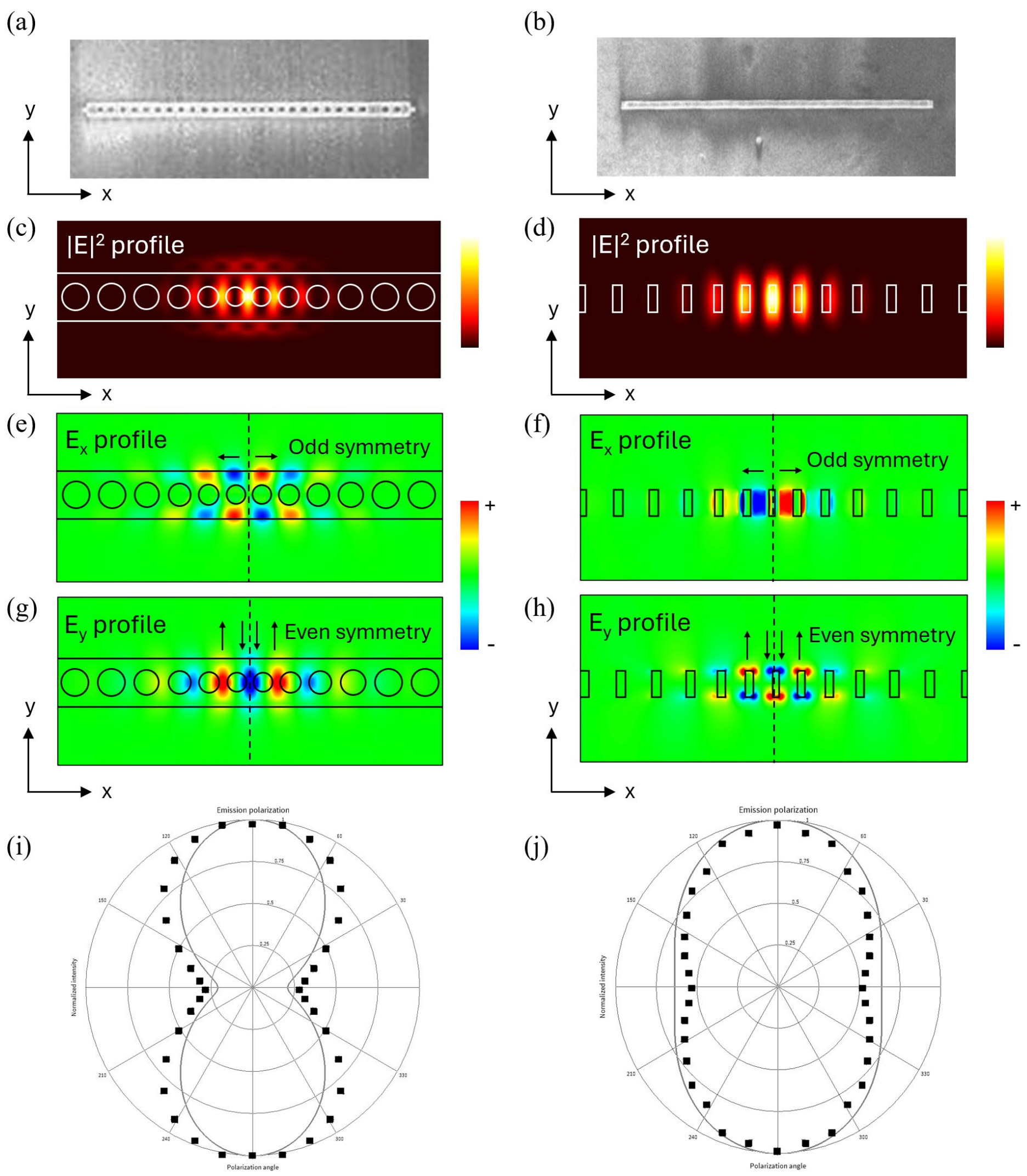

Figure S10. Cavity-mode symmetry and polarization of vertical emission from the P-PhC and R-PhC configurations. (a,b) Experimental images of the fabricated P-PhC and R-PhC nanobeam devices, respectively. (c,d) Calculated electric-field intensity distributions $|E|^2$ in the central vertical plane of the P-PhC and R-PhC cavities. (e,f) Real parts of the E_x field component and (g,h) Real parts of the E_y field component for the P-PhC and R-PhC configurations, respectively. The vertical dashed lines indicate the central mirror plane at $x = 0$. In both configurations, E_x exhibits odd symmetry whereas E_y exhibits even symmetry with respect to this plane. The odd-symmetric E_x contribution tends to cancel in the vertically emitted far field, while the even-symmetric E_y contribution adds constructively, resulting in predominantly y-polarized cavity emission. (i,j) Comparison of measured polarization responses (symbols) and calculated polarization characteristics (solid lines) for the P-PhC and R-PhC vertical cavity emission.

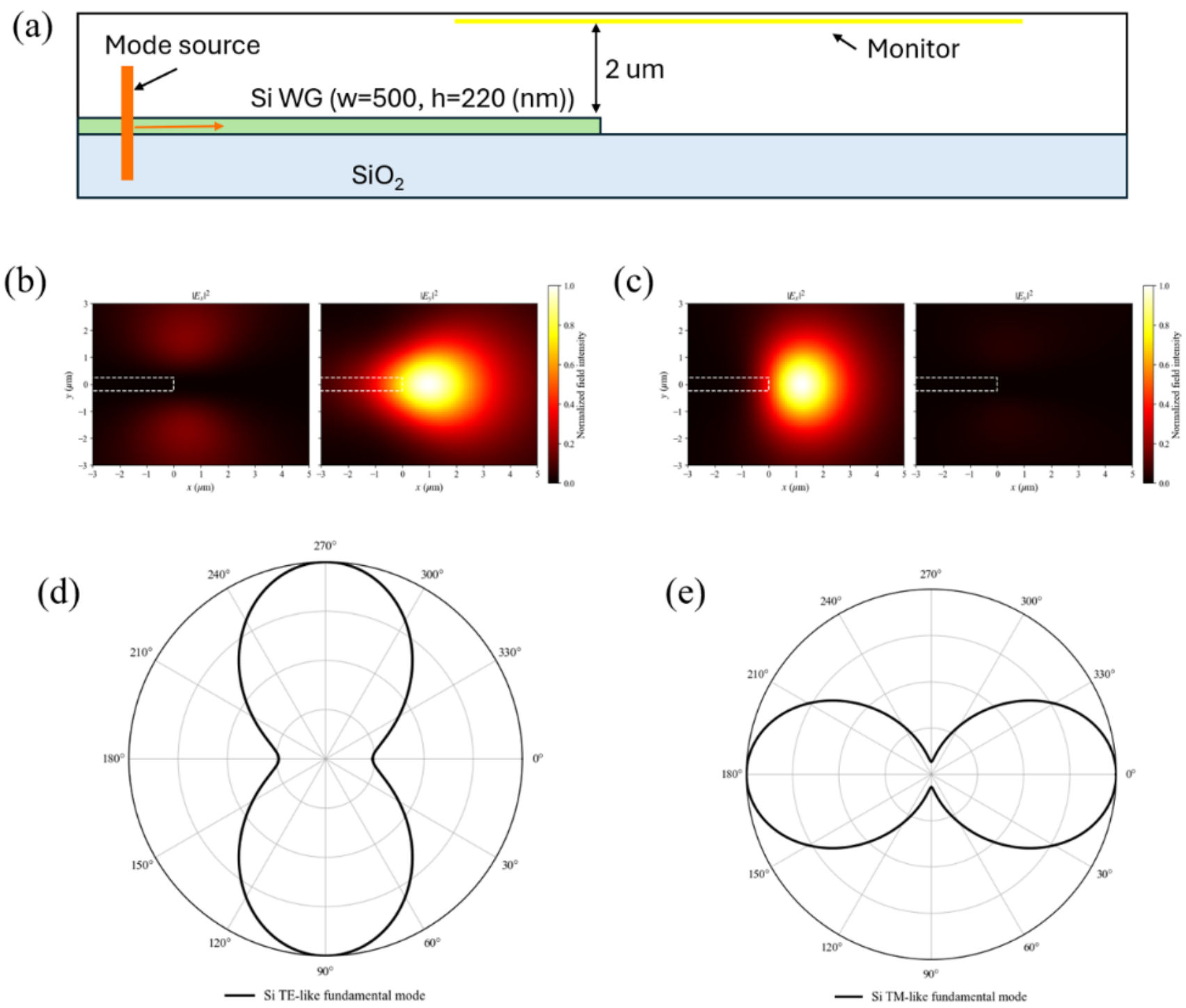

Figure S11. Polarization characteristics of vertically scattered emission from TE-like and TM-like silicon-waveguide modes. (a) Simulation geometry used to analyze vertical radiation from the silicon-waveguide termination. The fundamental guided mode is launched along the waveguide and the emitted field is recorded on a monitor positioned 2 µm above the structure. (b,c) Calculated transverse electric-field intensity distributions of the vertically scattered radiation generated by the TE-like and TM-like guided modes, respectively. (d,e) Corresponding polarization responses of the emitted beams. The TE-like guided mode produces predominantly y-polarized vertical radiation, whereas the TM-like guided mode produces predominantly x-polarized vertical radiation at the waveguide termination.

Supplementary Video. Rolling-Enabled Transfer-Printing Process for Controlled Rotation of a PhC Nanobeam

The video shows real-time optical monitoring of the rolling process using transparent PDMS stamps. Changes in optical contrast during contact and separation provide visual feedback of nanobeam rotation and enable controlled reorientation prior to final transfer printing.
